# A nationwide cohort study on growth impairment by cleft lip with or without palate

**DOI:** 10.1038/s41598-021-03052-x

**Published:** 2021-12-08

**Authors:** Jeong Yeop Ryu, Tae Hyun Park, Joon Seok Lee, Jung Dug Yang, Ho Yun Chung, Byung Chae Cho, Kang Young Choi

**Affiliations:** 1 Department of Plastic and Reconstructive Surgery, School of Medicine, Kyungpook National University, 680 Gukchaebosanro, Jung-gu, Daegu, 41405 Republic of Korea; 2 Cell and Matrix Research Institute, School of Medicine, Kyungpook National University, 680 Gukchaebosanro, Jung-gu, Daegu, 41405 Republic of Korea

**Keywords:** Outcomes research, Paediatric research

## Abstract

There are very few nationwide studies discussing the height, weight, head circumference, and dental screening of children with cleft lip with or without palate (CL/P), with most reports on this subject based on a 1900s cohort. This study aimed to characterize CL/P children in the 2000s in terms of height, weight, head circumference, and dental screening. This nationwide population-based study evaluated the National Health Insurance Service-Infants and Children’s Health Screening (NHIS-INCHS), specifically the height, weight, and head circumference of millions of children. Dental screening data, including the status of each tooth and comprehensive dental judgment, were also evaluated. Syndromic and nonsyndromic CL/P children had lower height, weight, and head circumference than no CL/P children until the age of 66–71 months. Children with cleft palate only or both cleft lip and palate showed similar results. Regarding dental screening, the primary teeth of CL/P children erupted later and fell out faster than no CL/P children. Dental caries was also more common in CL/P children. Children with CL/P had inferior general growth, regardless of palatoplasty surgery. More aggressive dental treatment was required for CL/P children due to the instability of primary teeth and tendency for caries.

## Introduction

Cleft lip with or without palate (CL/P) occurs in 1 out of 700 children, although the incidence varies by region and race^1^. CL/P infants have difficulty generating negative pressures for sucking and swallowing and thus have problems in breastfeeding and weaning^2^. Although there are specialized bottles for cleft palate infants, such as the Haberman feeder and Mead–Johnson feeder^3^, the maintenance of weight gain and skeletal growth of CL/P children is not well studied.

Comparative studies on weight gain and skeletal growth in CL/P children have been reported during 1980–2010. These reports show no difference in mean body weight at birth between infants with and without CL/P. However, in early infancy, CL/P infants showed lower weight gain than those without CL/P, and growth retardation was observed during the period of surgical lip repair. However, weight gain reportedly returned to normal after cleft palate repair, and the differences were evened out between CL/P and no CL/P children^3–8^. According to statistics released by the Korean Ministry of Education, children and adolescents aged 6–17 years gained more height in the 2000s than in the 1990s^9^. Although there are no growth data for infants and children aged 0–5 in this statistic, our authors inferred those infants and children aged 0-5 would have similar results. Therefore, we wanted to find out how the skeletal growth of CL/P infants and children in the 2000s differs from those of no CL/P infants and children. Since previous findings of comparative studies were reported before the 2000s, they do not represent the current situation well. These reports also did not study enough subjects to make a generalization about the growth of children. Currently, there is no nationwide population-based study on the relationship between CL/P and height, weight, and head circumference.

CL/P children differ from normal children in terms of oral and dental health. It is generally known that children with CL/P have restricted midface growth. Some recent papers reported on midface growth in relation to cleft palate surgery method and timing and found that malocclusion is more common in CL/P children^10,11^. However, there are few nationwide population-based studies making a comprehensive dental evaluation, including the speed of dental eruption, risk of tooth caries, and timing of permanent tooth eruption.

Herein, we investigated the general growth (height, weight, head circumference) and dental growth of CL/P children compared with age- and sex-matched control subjects.

## Methods

### Ethical approval

This study was approved by the Institutional Review Board of Kyungpook National University Hospital (IRB No. KNUH 2020-04-049) and performed in accordance with the principles of the Declaration of Helsinki. All personal information was anonymized. Disclosure and sharing of anonymized health insurance data were guaranteed by Korean law, and there was no reason to presume that participants refuse to consent. Because all data was anonymized, the risk of the study due to the waiver of consent was extremely low. Therefore, informed consents for participants waiver were obtained from Institutional Review Board of Kyungpook National University Hospital and Deliberation Committee of National Health Insurance Sharing Service in Korea.

### Data source

This cohort study was based on the Korean National Health Insurance (NHI) database. All Korean citizens, more than 50,000,000 people, are obliged to subscribe to the NHI system operated by the Korean government. This health insurance subscription is automatically made at the time of birth. Currently, 97% of Korean citizens are enrolled in this insurance system. The NHI database provides various medical inpatient and outpatient service usage. Information based on the International Classification of Diseases 10th revision (ICD-10) codes, diagnostic codes with treatments, all data on inpatient and outpatient claims, and sociodemographic data of all insured citizens were included in this database^12^. The National Health Insurance Service-Infants and Children’s Health Screening (NHIS-INCHS) started in 2007 in South Korea for all children under 6 years of age^13^.

### National health insurance service-infants and children’s health screening

All infants and children born in Korea in 2019 were eligible to participate in a total of seven children’s health screenings before entering elementary school (at 4–6, 9–12, 18–24, 30–36, 42–48, 54–60, 66–71 months of age). All infants and children born in Korea after 2020 underwent a total of eight screenings (a screening at 14–35 days of age was added). These screenings included the following: questionnaire filled out by the parents, examination by the pediatrician, physical measurement (height, weight, head circumference, BMI), health education, developmental screening, and counseling^14^. Moreover, since 2007, all infants and children born in Korea have undergone three oral and teeth examinations by a dentist (at 18–29, 42–53, 54–65 months of age). The dental screening included the current condition of each individual tooth (eruption, noneruption, erupting, demineralization, caries, restoration, pit and fissure sealing, suspicious caries), need for treatment, prevention, plaques, malocclusion, parafunction, caries, proximal caries, restoration, risk for caries, and total judgment of the teeth performed by a dentist. All dental records at 18–29 and 42–53 months were records of primary teeth (milk teeth), and dental records of 54–65 months included records of permanent teeth.

### Study population and subgroup analysis

**Figure 1:**
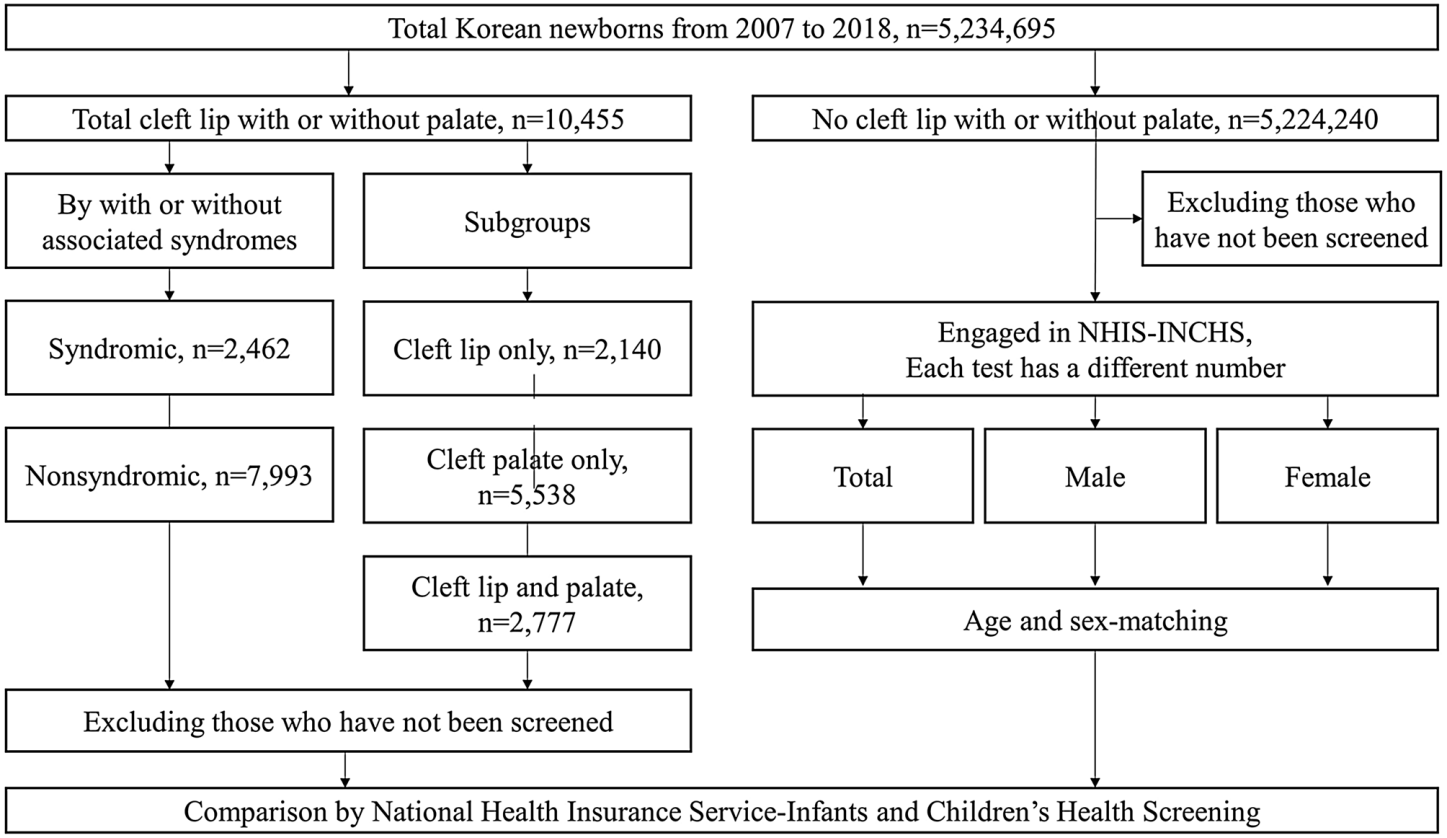
Flowchart of the study.

The 5,234,695 newborns born in 2007–2018 in Korea were classified into CL/P and control groups. The diagnosis of CL/P was identified based on ICD-10 codes (Q35–37). CL/P children were further divided into syndromic and nonsyndromic CL/P children based on the presence of associated syndromes. Detailed definitions of associated syndromes were described in Supplementary information. They were also divided into subgroups of cleft lip only (CLO), cleft palate only (CPO), and both cleft lip and palate (CLP) (Fig. 1).

### Validation for diagnostic accuracy

For diagnostic accuracy, a total of 437 children who had congenital facial anomalies and visited a single medical center from January 2006 to December 2018 were analyzed by reviewing the medical records. Two plastic surgeons reviewed all medical records, including medical photographs and 3-dimensional facial computed tomography (CT) findings. Cleft lips were identified by medical photographs and cheiloplasty (cleft lip repair). Cleft palates were diagnosed by medical photographs of the palate, 3-dimensional facial CT, and palatoplasty (cleft palate repair). To validate the subgroups of CL/P, CLO patients were defined as only those that were tagged for Q36 and not Q35. Cleft palate only (CPO) was tagged only with code Q35 and not Q36. Patients tagged with both Q35 and Q36 or Q37 were identified as cleft lip and palate (CLP). These algorithms were also used during the review of medical records in a single medical center.

Among all 437 children, 221 diagnosed with CL/P were analyzed for sensitivity, whereas 216 with facial congenital anomalies other than CL/P were analyzed for specificity. The sensitivity of these diagnostic algorithms was assessed by checking whether CL/P infants had satisfied the diagnostic criteria, and the specificity of these algorithms was assessed by determining why infants with congenital facial anomalies other than CL/P did not satisfy the diagnostic criteria. The results revealed that CL/P infants had 99.10% sensitivity and 99.07% specificity. The diagnostic algorithm for subgroup analysis showed 95.65% sensitivity and 97.83% specificity for CLO, 97.39% sensitivity and 99.13% specificity for CPO, and 96.67% sensitivity and 98.33% specificity for CLP.

### Statistical analysis

This study compared the general growth (i.e., height, weight, and head circumference) of CL/P and no CL/P children as well as compared their teeth by individual tooth number. These comparisons were performed according to sex, subgroups, and presence of associated syndromes. For general growth, all seven screenings were included in the analysis. Height, weight, and head circumference were divided into 1000 percentiles for the entire cohort. To exclude technical or input errors for each numerical value, numerical values less than the 1^st^ percentile and more than the 999th percentile were excluded. Mean values and standard deviation of the general growth data were calculated for CL/P and no CL/P children according to subgroups and the presence of associated syndromes. Analysis of variance with post hoc test (Scheffe) was used for statistical comparison. Regarding dental and oral examination, the differences regarding eruption, noneruption, erupting, demineralization, caries, restoration, pit and fissure sealing, and suspicious caries were compared. The categorical variables were analyzed using Chi-squared test. Categorical dependent variables, which included the needed treatment, prevention, plaque, malocclusion, parafunction, caries, proximal caries, restoration, risk for caries, and total judgment of the teeth, were also assessed. Logistic regression tests were performed for these categorical dependent variables. All statistical analyses were conducted using STATA MP, version 16.1 (StataCorp, College Station, Texas, USA). Statistical significance was set at *p* < 0.05.

## Results

A total of 5,234,695 newborns born during 2007–2018 were registered in the NHI database. Among them were 10,455 children with CL/P. There were 5292 males and 5163 females. There were 2462 cases of syndromic CL/P and 7993 cases of nonsyndromic CL/P. There were 2140, 5538, and 2777 children further classified into the CLO, CPO, and CLP subgroups, respectively (Table 1).

**Table 1:** Number of CL/P children during 2007–2018 in Korea.

	No. of children	Ratio (%)
Total	10,455	100
**Sex**		
Male	5292	50.62
Female	5163	49.38
**Presence of associated syndromes**		
Syndromic CL/P	2462	23.55
Nonsyndromic CL/P	7993	76.45
**Subgroups**		
CLO	2140	26.47
CPO	5538	52.97
CLP	2777	26.56
**Sensitivity for operation**		
Primary cheiloplasty	4820/4917 cases	98.03
Primary palatoplasty	8095/8315 cases	97.35

*CL/P* cleft lip with or without palate, *CLO* cleft lip only, *CPO* cleft palate only, *CLP*: cleft lip and palate.

### General growth (height, weight, and head circumference)

**Figure 2:**
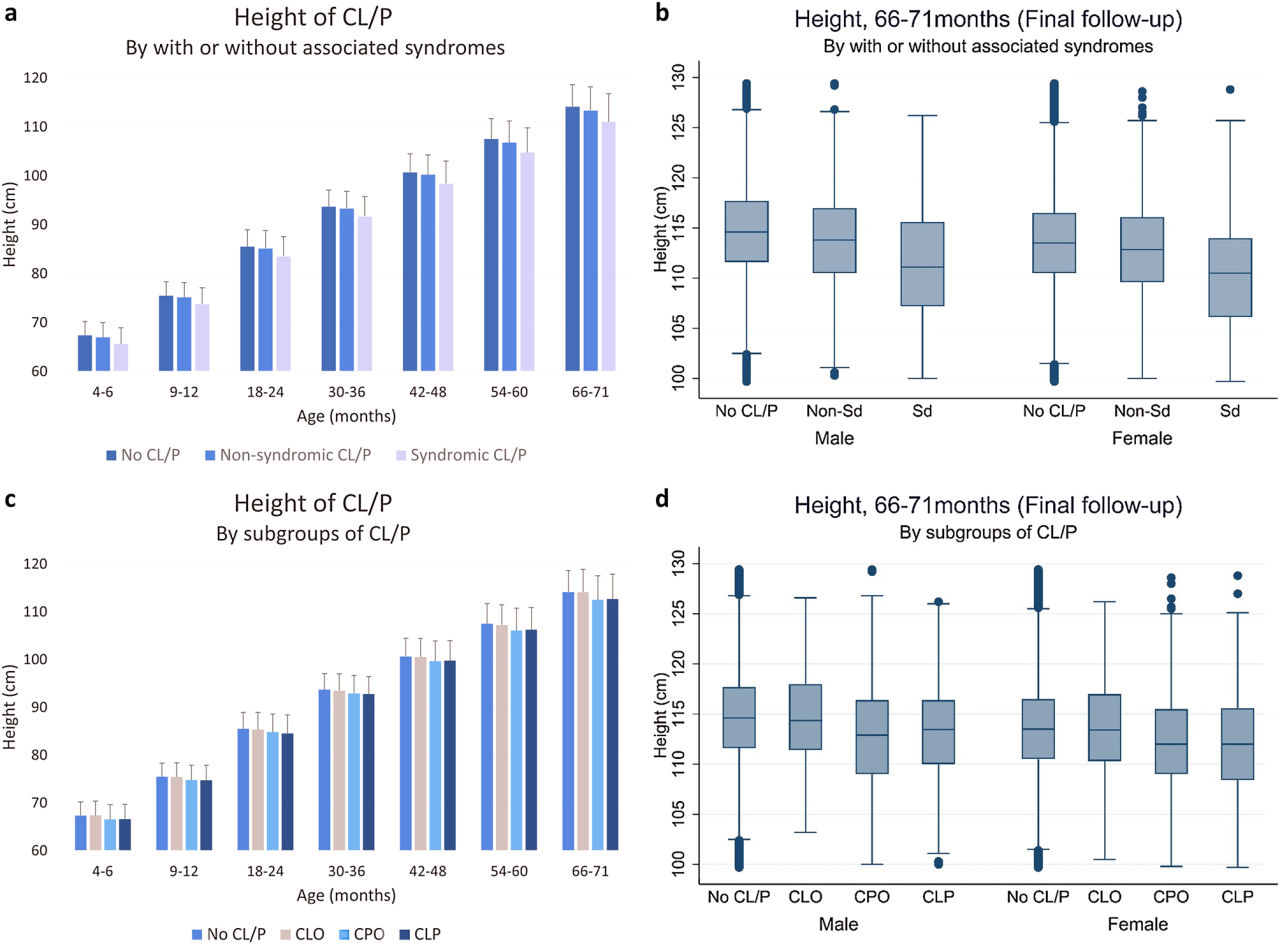
Comparison of height between CL/P and no CL/P children. (**a**) Height of CL/P children grouped by age and the presence of associated syndromes. (**b**) Height of CL/P children at 66–71 months grouped by the presence of associated syndromes. (**c**) Height of CL/P children grouped by age and subgroup (CLO, CPO, CLP). (**d**) Height of CL/P children at 66–71 months by subgroup (CLO, CPO, CLP).

Table 2 shows the mean heights of the study population analyzed via the post hoc test. At 66–71 months, the mean heights of nonsyndromic and syndromic CL/P children were lower than controls (by 0.78 cm and 3.13 cm, respectively). In the subgroup comparisons, there were no significant differences between CLO and controls and between CLP and CPO children in terms of height. However, at 66–71 months, CPO and CLP children were significantly shorter than controls (by 1.64 cm and 1.45 cm, respectively). This trend in height difference was consistent throughout all screenings (Fig. 2).

**Table 2:** Mean heights (cm) of CL/P and non-CL/P children during 2007–2018 in Korea.

Age (months)	No CL/P (a)	Presence of associated syndromes		Subgroups		
		N-Sd. (b)	Sd. (c)	CLO (d)	CPO (e)	CLP (f)
**4–6**						
Mean	67.36	66.99	65.62	67.40	66.54	66.61
Std. Dev.	2.84	2.98	3.27	2.97	3.08	3.08
Freq.	2,936,859	4631	1027	1225	3085	1348
**9–12**						
Mean	75.48	75.15	73.77	75.44	74.77	74.73
Std. Dev.	2.87	2.99	3.34	2.95	3.12	3.15
Freq.	3,090,339	4423	939	1192	2844	1326
**18–24**						
Mean	85.49	85.14	83.51	85.35	84.80	84.55
Std. Dev.	3.45	3.66	4.03	3.62	3.79	3.85
Freq.	3,342,769	5104	1085	1367	3224	1598
**30–36**						
Mean	93.69	93.30	91.69	93.49	92.92	92.79
Std. Dev.	3.37	3.51	4.05	3.51	3.73	3.64
Freq.	3,061,659	4744	1045	1251	2969	1569
**42–48**						
Mean	100.66	100.23	98.40	100.54	99.67	99.76
Std. Dev.	3.82	3.99	4.59	3.86	4.25	4.21
Freq.	2,597,652	4062	958	1088	2533	1399
**54–60**						
Mean	107.50	106.76	104.74	107.25	106.09	106.24
Std. Dev.	4.20	4.41	5.02	4.22	4.67	4.66
Freq.	2,092,872	3274	753	866	2008	1153
**66–71**						
Mean	114.13	113.35	111.00	114.13	112.49	112.68
Std. Dev.	4.50	4.80	5.78	4.80	5.06	5.18
Freq.	1,620,680	2553	583	705	1552	879
Post hoc test (Scheffe)	a>b>c			a, d > e, f		

The *p* values were omitted because all values were < 0.0001. The results of the post hoc test by Scheffe were described once because all the results were the same. *CL/P* cleft lip with or without palate, *N-Sd* nonsyndromic CL/P, *Sd* syndromic CL/P, *CLO* cleft lip only, *CPO* cleft palate only, *CLP* cleft lip and palate.

**Figure 3:**
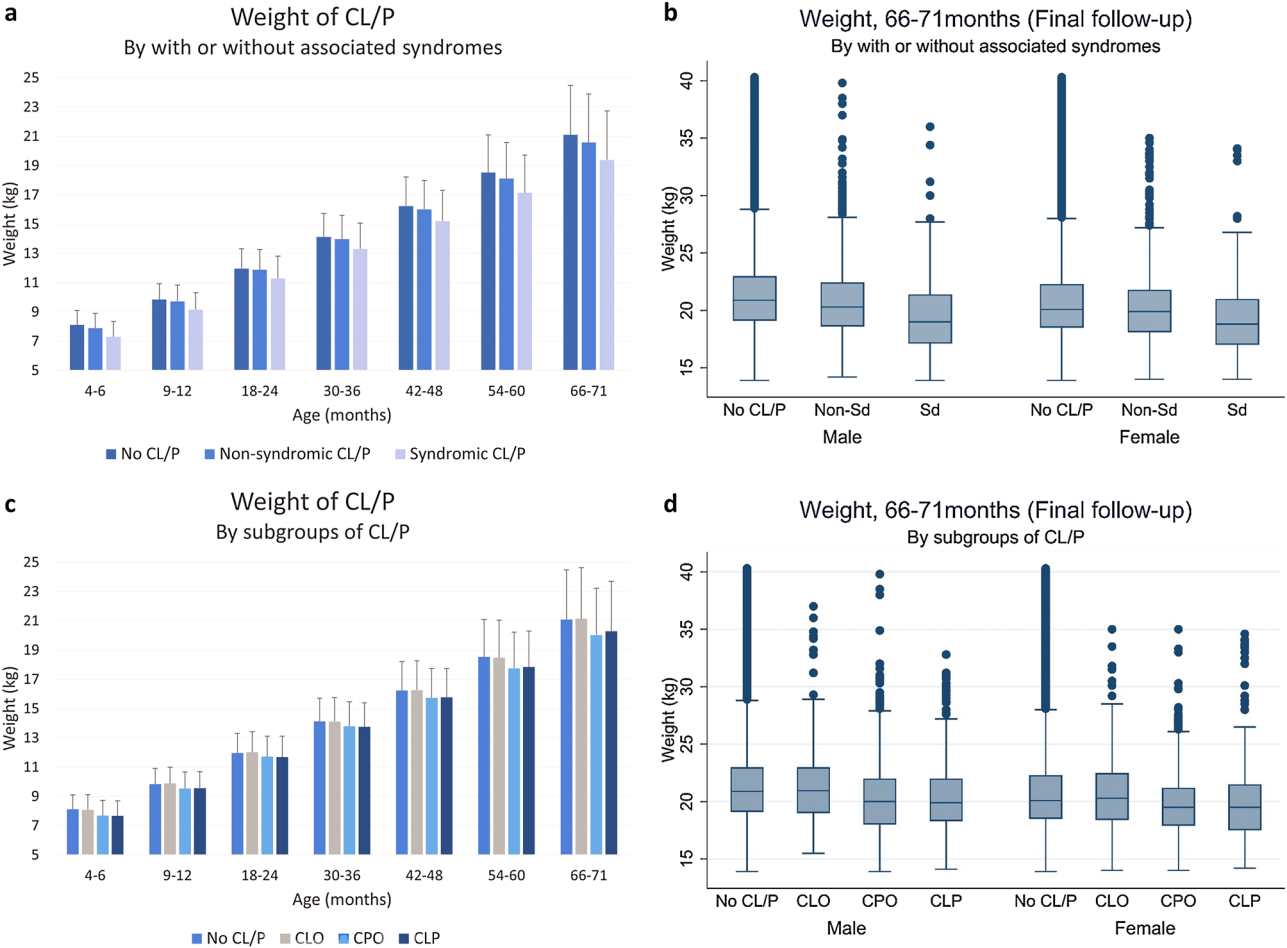
Comparison of weight between CL/P and no CL/P children. (**a**) Weight of CL/P children grouped by age and the presence of associated syndromes. (**b**) Weight of CL/P children at 66–71 months grouped by the presence of associated syndromes. (**c**) Weight of CL/P children grouped by age and subgroup (CLO, CPO, CLP). (**d**) Weight of CL/P children at 66–71 months by subgroup (CLO, CPO, CLP).

Table 3 shows the mean weights of the study population. At 66–71 months, nonsyndromic and syndromic CL/P children had lower mean weight than controls (by 0.52 kg and 1.71 kg, respectively). In the subgroup comparisons, there were no significant differences between CLO and controls and between CLP and CPO children. However, at 66–71 months, CPO and CLP children weighed significantly less than controls (by 1.06 kg and 0.81 kg, respectively). This trend in weight difference was consistent throughout all screenings (Fig. 3).

**Table 3:** Mean weights (kg) of CL/P and non-CL/P children during 2007–2018 in Korea.

Age (months)	No CL/P (a)	Presence of associated syndromes		Subgroups		
		N-Sd. (b)	Sd. (c)	CLO (d)	CPO (e)	CLP (f)
4–6						
Mean	8.11	7.88	7.29	8.09	7.69	7.67
Std. Dev.	0.99	1.01	1.06	1.03	1.04	1.01
Freq.	2,936,756	4610	994	1225	3043	1336
**9–12**						
Mean	9.84	9.72	9.16	9.90	9.53	9.56
Std. Dev.	1.08	1.10	1.15	1.09	1.13	1.12
Freq.	3,090,157	4413	922	1190	2824	1321
**18–24**						
Mean	11.96	11.88	11.29	12.01	11.71	11.69
Std. Dev.	1.34	1.37	1.51	1.39	1.41	1.43
Freq.	3,343,226	5111	1078	1365	3222	1602
**30–36**						
Mean	14.12	13.97	13.30	14.11	13.79	13.76
Std. Dev.	1.60	1.62	1.77	1.63	1.69	1.64
Freq.	3,062,165	4747	1044	1249	2969	1573
**42–48**						
Mean	16.23	16.01	15.21	16.26	15.73	15.76
Std. Dev.	1.97	1.96	2.10	2.00	2.02	1.98
Freq.	2,598,172	4067	957	1086	2537	1401
**54–60**						
Mean	18.52	18.11	17.13	18.47	17.75	17.83
Std. Dev.	2.57	2.45	2.58	2.57	2.47	2.46
Freq.	2,092,851	3278	750	864	2010	1154
**66–71**						
Mean	21.09	20.57	19.38	21.14	20.03	20.28
Std. Dev.	3.38	3.32	3.35	3.48	3.20	3.41
Freq.	1,620,589	2550	590	705	1558	877
Post hoc test (Scheffe)	a > b >c			a, d > e, f		

The *p* values were omitted because all values were < 0.0001. The results of the post hoc test by Scheffe were described once because all the results were the same. *CL/P* cleft lip with or without palate, *N-Sd* nonsyndromic CL/P, *Sd* syndromic CL/P, *CLO* cleft lip only, *CPO* cleft palate only, *CLP* cleft lip and palate.

**Figure 4:**
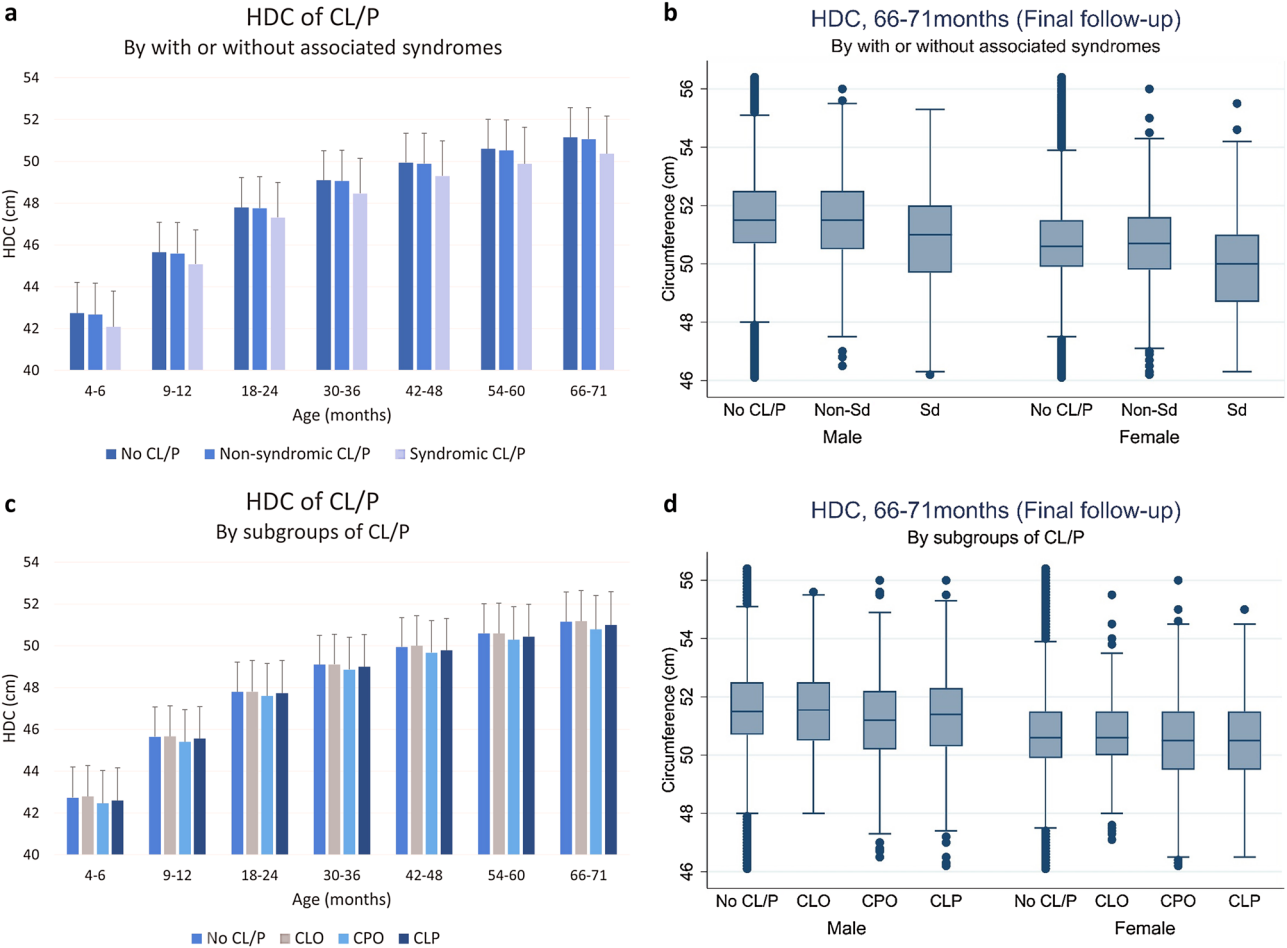
Comparison of head circumference between CL/P and no CL/P children. (**a**) Head circumference of CL/P children grouped by age and the presence of associated syndromes. (**b**) Head circumference of CL/P children at 66–71 months grouped by the presence of associated syndromes. (**c**) Head circumference of CL/P children grouped by age and subgroup (CLO, CPO, CLP). (**d**) Head circumference of CL/P children at 66–71 months by subgroup (CLO, CPO, CLP).

Table 4 shows the mean head circumferences of the study population. At the final screening, nonsyndromic and syndromic CL/P children had lower mean head circumference than controls (by 0.09 cm and 0.79 cm, respectively). During subgroup comparison, at 66–71 months, CLP children had lower mean circumference than controls, whereas CPO children had lower mean circumference than CLP children. CLO children and controls were not significantly different (Fig. 4).

**Table 4:** Mean head circumferences (cm) of CL/P and non-CL/P children during 2007–2018 in Korea.

Age (months)	No CL/P (a)	Presence of associated syndromes		Subgroups		
		N-Sd. (b)	Sd. (c)	CLO (d)	CPO (e)	CLP (f)
**4–6**						
Mean	42.73	42.67	42.09	42.80	42.46	42.59
Std. Dev.	1.47	1.50	1.70	1.47	1.57	1.57
Freq.	2,937,071	4623	1025	1226	3067	1355
Post hoc test (Scheffe)	a > b > c			a, d > e, f		
**9–12**						
Mean	45.65	45.59	45.08	45.67	45.40	45.56
Std. Dev.	1.43	1.48	1.63	1.45	1.55	1.53
Freq.	3,089,571	4423	950	1191	2851	1331
Post hoc test (Scheffe)	a > b > c			a, d, f > e		
**18–24**						
Mean	47.79	47.76	47.32	47.80	47.60	47.73
Std. Dev.	1.43	1.50	1.67	1.49	1.55	1.56
Freq.	3,342,266	5110	1082	1367	3223	1602
Post-hoc test (Scheffe)	a, b > c			a, d, f > e		
**30–36**						
Mean	49.10	49.06	48.47	49.11	48.86	49.00
Std. Dev.	1.40	1.47	1.67	1.44	1.55	1.54
Freq.	3,061,804	4757	1047	1251	2979	1574
Post hoc test (Scheffe)	a, b > c			a, d, f > e		
**42–48**						
Mean	49.94	49.88	49.30	50.01	49.67	49.78
Std. Dev.	1.40	1.45	1.68	1.43	1.53	1.53
Freq.	2,597,603	4070	966	1087	2550	1399
Post hoc test (Scheffe)	a > b > c			a, d > e, f		
**54–60**						
Mean	50.59	50.52	49.88	50.59	50.29	50.44
Std. Dev.	1.41	1.46	1.75	1.44	1.58	1.54
Freq.	2,093,408	3277	764	868	2020	1153
Post hoc test (Scheffe)	a > b > c			a > f > e; d > e		
**66–71**						
Mean	51.15	51.06	50.36	51.17	50.79	50.99
Std. Dev.	1.42	1.50	1.80	1.46	1.62	1.59
Freq.	1,620,429	2557	590	703	1564	880
Post hoc test (Scheffe)	a > b > c			a > f > e; d > e		

The *p* values were omitted because all values were < 0.0001. *CL/P* cleft lip with or without palate, *N-Sd* nonsyndromic CL/P, *Sd* syndromic CL/P, *CLO* cleft lip only, *CPO* cleft palate only, *CLP* cleft lip and palate.

### Dental and oral screening

All infants and children born in Korea have undergone three dental and oral examinations by a dentist (at 18–29, 42–53, 54–65 months of age) under the policy of NHI. Therefore, the results of dental and oral examination were present only in these three periods contrary to the general health screenings. These screenings were compared between no CL/P and CL/P children (Supplementary Table 2–25). CL/P children had slower eruption of primary teeth than no CL/P children, except for the primary second molars (#55, #65, #75, #85). However, all the permanent teeth of CL/P children erupted faster than controls.

CL/P primary teeth had more demineralization, caries, and restoration than controls, except for the upper primary incisors (#51, #52, #61, #62), wherein they had less demineralization and caries, but higher restorations than controls. Compared to controls, CL/P children had no difference in the upper central incisors (#11, #12), but increased demineralization, caries, and reduced restoration in the lower central incisors (#31, #41). There was increased caries, reduced demineralization, and reduced restoration in the lateral incisors (#12, #22), and increased caries, increased demineralization, and reduced restoration in the lower lateral incisors (#32, #42). The first upper teeth molars (#16, #26) also had increased demineralization, increased caries, and decreased restoration. The first lower teeth molars (#36, #46), were not different between CL/P and no CL/P children. The statistical differences of each tooth between CL/P and no CL/P children are shown in both the upper and lower teeth (Supplementary Table 26).

CL/P children had significantly increased risks for malocclusion, parafunction, caries, risk for caries, and poor total judgment than controls at 18–29 months. At 42–53 months, CL/P children had increased risk for malocclusion and parafunction but decreased risk for needing treatment. At 54–65 months, CL/P children had increased risk for malocclusion, caries, restoration, and poor total judgment and a decreased risk for needing treatment (Table 5).

**Table 5:** Logistic regression analysis of the dental examinations of CL/P children during 2007–2018 in Korea.

Age (months)	Dependent variables	OR (95% CIs)		*P*	Number of obs
		No CL/P	CL/P		
18–29	Tx needed	Reference	0.90 (0.77–1.06)	0.214	658,805
	Pv needed	Reference	0.93 (0.83–1.05)	0.220	658,807
	Plaque	Reference	0.91 (0.80–1.02)	0.111	658,805
	Malocclusion	Reference	2.50 (2.15–2.91)	< 0.0001***	658,798
	Parafunction	Reference	2.64 (1.12–6.27)	0.027*	1,110,318
	Caries	Reference	1.16 (1.03–1.30)	0.014*	1,114,129
	Caries, prox	Reference	1.05 (0.86–1.27)	0.662	1,114,130
	Restoration	Reference	1.18 (0.80–1.75)	0.411	1,114,125
	Caries risk	Reference	4.22 (1.73–10.28)	0.002**	1,112,628
	Total judg- NG	Reference	1.09 (1.01–1.17)	0.029*	1,620,973
42–53	Tx needed	Reference	0.84 (0.72–0.98)	0.031*	586,093
	Pv needed	Reference	1.00 (0.85–1.17)	0.991	586,088
	Plaque	Reference	1.02 (0.87–1.20)	0.812	586,090
	Malocclusion	Reference	3.58 (2.89–4.44)	< 0.0001***	586,089
	Parafunction	Reference	1.67 (1.08–2.58)	0.021*	586,086
	Caries	Reference	1.07 (0.98–1.18)	0.138	1,076,685
	Caries, prox	Reference	1.02 (0.91–1.15)	0.721	1,076,677
	Restoration	Reference	1.08 (0.95–1.22)	0.253	1,076,673
	Caries risk	Reference	1.04 (0.94–1.15)	0.445	1,076,670
	Total judg- NG	Reference	1.17 (1.06–1.29)	0.002**	1,092,390
54–65	Tx needed	Reference	0.76 (0.63–0.92)	0.005**	464,682
	Pv needed	Reference	0.97 (0.80–1.17)	0.717	464,681
	Plaque	Reference	1.07 (0.88–1.31)	0.481	464,681
	Malocclusion	Reference	4.14 (3.20–5.36)	< 0.0001***	464,680
	Parafunction	Reference	1.57 (0.88–2.78)	0.125	464,678
	Caries	Reference	1.20 (1.08–1.33)	0.001**	894,963
	Caries, prox	Reference	0.97 (0.86–1.10)	0.675	894,957
	Restoration	Reference	1.12 (1.00–1.26)	0.048*	894,954
	Caries risk	Reference	1.08 (0.96–1.20)	0.208	894,952
	Total judg-NG	Reference	1.33 (1.18–1.49)	< 0.0001***	894,070

*P < 0.05, **P < 0.01, ***P < 0.001, *OR* odds ratio, *CI* confidence interval, *obs* observations, *Tx* treatment, *Pv* prevention, *prox* proximal, *judg* judgment, *NG* not good, *CL/P* cleft lip with or without palate.

## Discussion

This study compared the general growth and orodental conditions of CL/P and no CL/P children. Both syndromic and nonsyndromic CL/P children were generally shorter than controls at any age. At the final follow-up (66–71 months), nonsyndromic and syndromic CL/P children were shorter than controls. At 66–71 months, nonsyndromic and syndromic CL/P children weighed less than no CL/P children. The CL/P children never had more height or weight than no CL/P children.

At 66–71 months, nonsyndromic CL/P children were shorter by <1 cm, but syndromic CL/P children were shorter by >3 cm, indicating a big difference. Syndromic CL/P is closely correlated with chromosomal anomalies, and these patients may have developmental delay, cardiac anomalies, or a severely shortened life span^3^. Thus, these growth disorders in syndromic CL/P children may have been caused by their associated congenital anomalies and intrinsic factors. Interestingly, children with nonsyndromic CL/P also had lower height and weight, but they did not differ in intrinsic factors. Thus, the difference may be due to the environmental factors caused by CL/P. However, this raises a few questions: (1) What is the normal height? (2) Can nonsyndromic children 0.78 cm shorter than no CL/P children be considered abnormal? Since the average height did not differ by even 1 cm, it is difficult to consider nonsyndromic CL/P children as abnormal. Nevertheless, it was a clear finding that nonsyndromic CL/P children are shorter than no CL/P children. According to previous reports, CL/P and no CL/P children were not different at birth, but growth retardation appeared in CL/P children upon reaching the appropriate age for undergoing palatoplasty. Moreover, after palatoplasty, growth was recovered at 4 years, and the difference with no CL/P children disappeared. CL/P and no CL/P children did not differ in growth by 6 years of age. However, in late childhood, CL/P children had a greater average decrease in height and weight^3^.

On subgroup analysis, CLO and CL/P children were not different from controls, but those with CPO or CLP had lower height and weight until 66–71 months of age. According to previous study which was regarding the length and body weight of millions of newborns including CL/P infants, compared to controls, the body dimensions of infants with CLO (n = 865) were not different at birth, whereas those with CPO and CLP had lower weight and height. However, in that study, long-term follow-up was lacking, and the subjects were infants born during 1973–1992^6^. In our study, most cases (8095 out of 8315 cases; 97.35%) requiring palatoplasty underwent primary palatoplasty. Conversely, our study found that growth in terms of height and weight could not catch up with that of normal children after palatoplasty surgery.

In dental and oral screenings, primary teeth of CL/P children erupted slower than those without CL/P, except for the primary second molars (#55, #65, #75, #85). However, all permanent teeth of CL/P children erupted faster than those without CL/P. The rapid eruption of the permanent teeth means that the primary teeth were quickly dislodged, suggesting greatly reduced stability in the primary teeth of CL/P children. Interestingly, even though CL/P is associated with the upper teeth, similar results were found in the lower teeth of CL/P children. Some believe that these delays in growth are because many CL/P infants are born prematurely^15^. Although we did not describe this in the results section, in our cohort, only 4% of no CL/P children and 7% of CL/P children were premature. Furthermore, if these results were obtained because there were many premature infants among those with CL/P, the permanent teeth would have erupted later than in those without CL/P.

In our study, CL/P children never caught up with the growth of no CL/P children, except in head circumference. Unfortunately, the current evidence based on textbook descriptions is from a population cohort in the 1900s, and thus it is not representative of the recent growth of children. Because “catch up” is a relative concept, no matter how well CL/P children eat after palatoplasty, catch up growth can still be demonstrated if no CL/P children in the 2000s have better growth indicators than those in the 1900s. Moreover, although efforts are made to improve feeding in children with cleft palate, these may still be insufficient in early infancy, especially because nonsyndromic cleft palate infants do not differ in digestive ability compared to normal infants. The low levels of general growth in syndromic and nonsyndromic CL/P, CPO, and CLP children suggest anatomical deficiencies in oral intake. Therefore, we believe that short-term tube feeding assistance or palatal splits may be helpful for increasing oral intake during early infancy.

On dental examination, CL/P children had higher risks than CL/P children in terms of malocclusion, parafunction, caries, and risk of caries at 18–29 months. In contrast, at 42–53 months, CL/P children had a higher risk than no CL/P children in terms of malocclusion and parafunction only, but not in caries or risk of caries. Additionally, CL/P children needed less dental treatments than no CL/P children, which is likely because they had more frequent dental visits before 42–53 months of age and received dental care more quickly. There also seems to be a difference in sensitivity to dental treatment. Similarly, at 54–65 months, CL/P children had a lower risk of requiring treatment, but a higher risk of malocclusion and caries. Dental restorations that had already been performed were also more common in CL/P. This also supports the high sensitivity of CL/P children to dental treatment. In all age groups, CL/P children had higher rates of malocclusion and poor total judgment, and the odds ratio for these two variables also gradually increased with age. This suggests that CL/P children should receive dental care more actively before entering elementary school.

This study has some limitations. Since diagnoses were based using the ICD-10 code, detailed diagnostic classifications reflecting severity (i.e., unilateral and bilateral CL/P) were not available. Medical images, photographs, radiologic findings, or laboratory findings were also not included in this database. Thus, selection bias from diagnostic classification may exist. However, our authors validated the diagnostic accuracy of the ICD-10 codes. The Korean NHI is a fairly accurate database as well; the insurance review teams of each general hospital in Korea verify the ICD-10 code and surgical fees before hospitals claim medical fees. Afterwards, the Health Insurance Review & Assessment, which is another national public institution separate from the NHI, performs reverification. Our data from the Korean NHI went through these two steps of verification. Therefore, the bias for misdiagnosis in the present study is low.

This study also has some strengths. First, this is the first comparative report to investigate the nationwide general growth, dental growth, and dental examinations in Asian CL/P children, to the best of our knowledge. Second, because this study included children during 2007–2018, it reflects the recent growth of children.

## Conclusion

CL/P children had lower height, weight, and head circumference than no CL/P children before elementary school with or without surgery during 2007–2018. Both syndromic and nonsyndromic CL/P had similar results. These values were also lower in CPO and CLP children, whereas no difference was found between CLO and no CL/P children. All primary teeth in CL/P children, except the second molars, erupted later than in those without CL/P. Contrarily, permanent teeth in CL/P children erupted faster than in those without CL/P, which means that CL/P children lose their primary teeth more quickly. Moreover, CL/P children had a higher risk for caries and malocclusion than those without CL/P and hence need to receive more aggressive dental treatment before entering elementary school.

## Supplementary Information

Supplementary Information.

## Data Availability

The data that supports the findings of this study is available from the NHI service, but restrictions apply to the availability of data, which was used with permission for the current study and therefore not publicly available. Data is however available upon reasonable request and with permission of NHI.
