# Supplementary material for: A nationwide cohort study on growth impairment by cleft lip with or without palate
Source: Sci Rep. 2021 Dec 8;11:23609. doi: 10.1038/s41598-021-03052-x (PMC8655029; doi:10.1038/s41598-021-03052-x)
Supplement: Supplementary file 1 — Supplementary Information. [file 41598_2021_3052_MOESM1_ESM.docx]

**Supplementary Information**

A nationwide cohort study on growth impairment by cleft lip with or without palate

Jeong Yeop Ryu MD, PhD^1^, Tae Hyun Park MD^1^, Joon Seok Lee MD, PhD^1^,

Jung Dug Yang, MD, PhD^1^, Ho Yun Chung, MD, PhD^1,2^, Byung Chae Cho MD, PhD^1^,

and Kang Young Choi MD, DMD, PhD^1^

^1^Department of Plastic and Reconstructive Surgery, ^2^Cell & Matrix Research Institute,

School of Medicine, Kyungpook National University,

680 Gukchaebosanro, Jung-gu, Daegu 41405, Republic of Korea

**Supplementary Methods**

**Definition of associated syndromes**

Congenital malformation syndromes that predominantly affect facial appearance (Q87.0), such as acrocephalopolysyndactyly, acrocephalosyndactyly (Apert), cryptophthalmos syndrome, cyclopia, Goldenhar syndrome, Gorlin–Chaudhry–Moss syndrome, Moebius syndrome, oro-facial-digital syndrome, Pierre–Robin syndrome, and whistling face, were all included. Velocardiofacial syndrome, which is caused by chromosome 22q11.2 deletion, results in pharyngeal dysfunction, cardiac anomalies, and facial dysmorphia.^1^ In the CL/P cohort, children identified with codes for congenital cardiac anomalies were categorized as children diagnosed with velocardiofacial syndrome. Since they can also be diagnosed with DiGeorge syndrome, the code for DiGeorge syndrome (D82.1) was also used to identify velocardiofacial syndrome. Mandibulofacial dysostosis (Q75.4) included Franceschetti syndrome and Treacher Collins syndrome. Nager syndrome is characterized by craniofacial and upper limb abnormalities. Acrofacial dysostosis manifests as craniofacial abnormalities in Nager syndrome and exhibits upper limb abnormalities.^2^ Cases with a combination of craniofacial dysostosis (Q75.1) or mandibulofacial dysostosis (Q75.4) with upper limb abnormalities (Q69 or Q70 or Q71) were identified as having Nager syndrome. Other specified congenital malformation syndromes (Q87.8) included Alport syndrome, Laurence–Moon(–Bardet)–Biedl syndrome, Zellweger syndrome, and CHARGE associations. VACTERL association is typically defined by the presence of three of the following congenital malformations: vertebral defects, anal atresia, cardiac defects, tracheoesophageal fistula, renal anomalies, and limb abnormalities.^3^ Vertebral defects (Q67.5, Q76.0, Q76.1–Q76.4), anal atresia (Q42.2, Q42.3), cardiac defects (Q20–Q26), tracheoesophageal fistula (Q39.1, Q39.2), renal anomalies (Q60–Q64), and limb abnormalities (Q65–Q74) were identified by ICD-10 codes. When three or more than three malformations were identified among them, VACTERL associations were defined. Congenital malformation syndromes predominantly associated with short stature (Q87.1) included Aarskog, Cockayne, De Lange, Dubowitz, Noonan Prader–Willi, Robinow–Silverman–Smith, Russel–Silver, Seckel, and Smith–Lemli–Opitz syndromes. Finally, congenital malformations, deformations, and chromosomal abnormalities (Q00–Q99) included Down, Edwards, and Patau syndromes, trisomies, monosomies, and chromosomal rearrangement, Turner’s syndrome, sex chromosome abnormalities, and other chromosomal abnormalities (Supplementary Table 1). When CL/P infants had one of above syndromes, they were defined as having syndromic CL/P.

**Supplementary References**

1 Kobrynski LJ, Sullivan KE. Velocardiofacial syndrome, DiGeorge syndrome: The chromosome 22q11.2 deletion syndromes. Lancet. 2007;370:1443-52.

2 Chummun S, McLean NR, Anderson PJ, Nieuwenhoven Cv, Mathijssen I, David DJ. The craniofacial and upper limb management of Nager syndrome. J Craniofac Surg. 2016;27:932-7.

3 Solomon BD. VACTERL/VATER Association. Orphanet J Rare Dis. 2011;6:56. doi: 10.1186/1750-1172-6-56.

**Supplementary Table 1. Definitions of associated syndromes with CL/P using ICD-10 codes.**

| Congenital malformation syndromes predominantly affecting facial appearance (Q87.0) | |  | VACTERL association | |
| --- | --- | --- | --- | --- |
| Q870.001 | Acrocephalopolysyndactyly |  | Q76.3 | Congenital scoliosis due to congenital bony malformation |
| Q870.002 | Acrocephalosyndactyly (Apert) |  | Q42.3 | Congenital absence, atresia and stenosis of anus without fistula |
| Q870.004 | Cryptophthalmos syndrome |  | Q20 | Congenital malformations of cardiac chambers and connections |
| Q870.005 | Cyclopia |  | Q21 | Congenital malformations of cardiac septa |
| Q870.010 | Whistling face |  | Q22 | Congenital malformations of pulmonary and tricuspid valves |
| Q870.006 | Goldenhar syndrome |  | Q23 | Congenital malformations of aortic and mitral valves |
| Q870.023 | Gorlin-Chaudhry-Moss syndrome |  | Q24 | Other congenital malformations of heart |
| Q870.007 | Moebius syndrome |  | Q25 | Congenital malformations of great arteries |
| Q870.008 | Oro-facial-digital syndrome |  | Q26 | Congenital malformations of great veins |
| Q870.009 | Robine syndrome |  | Q39.2 | Congenital tracheo-oesophageal fistula without atresia |
| Q870.014 | Pierre Robin sequence |  | Q60 | Renal agenesis and other reduction defects of kidney |
| Q870.031 | Stickler syndrome |  | Q61 | Cystic kidney disease |
|  |  |  |  |  |
| Velocardiofacial syndrome (Q35-37 + Q20-26, or D82.1) | |  | Q62 | Congenital obstructive defects of renal pelvis and congenital malformations of ureter |
|  |  |  |  |  |
| Q35 | Cleft palate |  | Q63 | Other congenital malformations of kidney |
| Q36 | Cleft lip |  | Q64 | Other congenital malformations of urinary system |
| Q37 | Cleft lip and cleft palate |  | Q65 | Congenital deformities of hip |
| Q20 | Congenital malformations of cardiac chambers and connections |  | Q66 | Congenital deformities of feet |
| Q21 | Congenital malformations of cardiac septa |  | Q67 | Congenital musculoskeletal deformities of head, face, spine and chest |
| Q22 | Congenital malformations of pulmonary and tricuspid valves |  | Q68 | Other congenital musculoskeletal deformities |
| Q23 | Congenital malformations of aortic and mitral valves |  | Q69 | Polydactyly |
| Q24 | Other congenital malformations of heart |  | Q70 | Syndactyly |
| Q25 | Congenital malformations of great arteries |  | Q71 | Reduction defects of upper limb |
| Q26 | Congenital malformations of great veins |  | Q72 | Reduction defects of lower limb |
| D82.1 | Di George’s syndrome |  | Q73 | Reduction defects of unspecified limb |
|  |  |  | Q74 | Other congenital malformations of limb(s) |
| Mandibulofacial dysostosis (Q75.4) | |  | Congenital malformation syndromes predominantly associated with short stature (Q87.1) | |
|  |  |  |  |  |
| Q754.001 | Franceschetti syndrome |  |  |  |
| Q754.000.01 | Treacher Collins syndrome |  |  | Aarskog |
|  |  |  |  | Cockayne |
| Nager syndrome (Q75.1 or Q75.4 + Q69 or Q70 or Q71) | |  |  | Cornelia de Lange |
| Q75.1 | Craniofacial dysostosis |  |  | Dubowitz |
| Q69 | Polydactyly |  |  | Noonan |
| Q70 | Syndactyly |  |  | Prader-Willi |
| Q71 | Reduction defects of upper limb |  |  | Robinow-Silverman-Smith |
|  |  |  |  | Russel-Silver |
| Other specified congenital malformation syndromes (Q87.8) | |  |  | Seckel |
|  | Alport syndrome |  |  | Smith-Lemli-Opitz |
|  | Laurence-Moon(-Bardet)-Biedl syndrome |  |  |  |
|  | Zellweger syndrome |  |  |  |
|  | CHARGE syndrome |  |  |  |
|  |  |  |  |  |
| Congenital malformations, deformations and chromosomal abnormalities | |  |  |  |
| Q90 | Down’s syndrome |  |  |  |
| Q91 | Edwards’ syndrome and Patau’s syndrome |  |  |  |
| Q92 | Other trisomies and partial trisomies of the autosomes |  |  |  |
| Q93 | Monosomies and deletions from the autosomes |  |  |  |
| Q95 | Balanced rearrangements and structural markers |  |  |  |
| Q96 | Turner’s syndrome |  |  |  |
| Q97 | Other sex chromosome abnormalities, female phenotype |  |  |  |
| Q98 | Other sex chromosome abnormalities, male phenotype |  |  |  |
| Q99 | Other chromosome abnormalities |  |  |  |

**Supplementary Table 2. Comparison of tooth #55 between CL/P and non-CL/P children by age group**

| Age (months) | 18–29 | | |  | 42–53 | | |  | 54–65 | | |
| --- | --- | --- | --- | --- | --- | --- | --- | --- | --- | --- | --- |
| Tooth #55 | No CL/P | CL/P | Total |  | No CL/P | CL/P | Total |  | No CL/P | CL/P | Total |
| Eruption | 1,202,194 | 2,268 | 1,204,462 |  | 1,008,776 | 1,861 | 1,010,637 |  | 723,504 | 1,303 | 724,807 |
| (%) | 68.16 | 68.71 | 68.16 |  | 82.17 | 81.34 | 82.17 |  | 77.59 | 74.25 | 77.58 |
| Noneruption | 522,363 | 951 | 523,314 |  | 1,166 | 2 | 1,168 |  | 355 | 1 | 356 |
| (%) | 29.62 | 28.81 | 29.61 |  | 0.09 | 0.09 | 0.09 |  | 0.04 | 0.06 | 0.04 |
| Erupting | 26,755 | 44 | 26,799 |  | 1,458 | 4 | 1,462 |  | 278 | 0 | 278 |
| (%) | 1.52 | 1.33 | 1.52 |  | 0.12 | 0.17 | 0.12 |  | 0.03 | 0.00 | 0.03 |
| Demineralization | 1,026 | 3 | 1,029 |  | 10,108 | 18 | 10,126 |  | 5,993 | 5 | 5,998 |
| (%) | 0.06 | 0.09 | 0.06 |  | 0.82 | 0.79 | 0.82 |  | 0.64 | 0.28 | 0.64 |
| Caries | 7,269 | 27 | 7,296 |  | 155,647 | 291 | 155,938 |  | 122,105 | 259 | 122,364 |
| (%) | 0.41 | 0.82 | 0.41 |  | 12.68 | 12.72 | 12.68 |  | 13.09 | 14.76 | 13.1 |
| Restoration | 2,298 | 6 | 2,304 |  | 40,696 | 98 | 40,794 |  | 71,599 | 174 | 71,773 |
| (%) | 0.13 | 0.18 | 0.13 |  | 3.31 | 4.28 | 3.32 |  | 7.68 | 9.91 | 7.68 |
| Pit and fissure sealing | 1,370 | 1 | 1,371 |  | 4,226 | 9 | 4,235 |  | 3,964 | 6 | 3,970 |
| (%) | 0.08 | 0.03 | 0.08 |  | 0.34 | 0.39 | 0.34 |  | 0.43 | 0.34 | 0.42 |
| Caries, suspicious | 509 | 1 | 510 |  | 5,629 | 5 | 5,634 |  | 4,721 | 7 | 4,728 |
| (%) | 0.03 | 0.03 | 0.03 |  | 0.46 | 0.22 | 0.46 |  | 0.51 | 0.40 | 0.51 |
| Total | 1,763,784 | 3,301 | 1,767,085 |  | 1,227,706 | 2,288 | 1,229,994 |  | 932,519 | 1,755 | 934,274 |
| (%) | 100 | 100 | 100 |  | 100 | 100 | 100 |  | 100 | 100 | 100 |
| P | 0.018* | | |  | 0.170 | | |  | 0.002** | | |

*P < 0.05, **P < 0.01, ***P < 0.001, CL/P: cleft lip with or without palate

**Supplementary Table 3. Comparison of tooth #54 between CL/P and non-CL/P children by age group**

| Age (months) | 18–29 | | |  | 42–53 | | |  | 54–65 | | |
| --- | --- | --- | --- | --- | --- | --- | --- | --- | --- | --- | --- |
| Tooth #54 | No CL/P | CL/P | Total |  | No CL/P | CL/P | Total |  | No CL/P | CL/P | Total |
| Eruption | 1,645,765 | 3,026 | 1,648,791 |  | 1,111,436 | 2,020 | 1,113,456 |  | 798,270 | 1,452 | 799,722 |
| (%) | 94.36 | 92.54 | 94.36 |  | 89.92 | 87.33 | 89.91 |  | 85.12 | 82.50 | 85.11 |
| Noneruption | 16,679 | 48 | 16,727 |  | 99 | 0 | 99 |  | 106 | 1 | 107 |
| (%) | 0.96 | 1.47 | 0.96 |  | 0.01 | 0.00 | 0.01 |  | 0.01 | 0.06 | 0.01 |
| Erupting | 27,931 | 56 | 27,987 |  | 252 | 0 | 252 |  | 185 | 2 | 187 |
| (%) | 1.6 | 1.71 | 1.6 |  | 0.02 | 0.00 | 0.02 |  | 0.02 | 0.11 | 0.02 |
| Demineralization | 9,051 | 22 | 9,073 |  | 4,183 | 5 | 4,188 |  | 2,360 | 5 | 2,365 |
| (%) | 0.52 | 0.67 | 0.52 |  | 0.34 | 0.22 | 0.34 |  | 0.25 | 0.28 | 0.25 |
| Caries | 40,472 | 108 | 40,580 |  | 76,799 | 181 | 76,980 |  | 72,358 | 156 | 72,514 |
| (%) | 2.32 | 3.30 | 2.32 |  | 6.21 | 7.83 | 6.22 |  | 7.72 | 8.86 | 7.72 |
| Restoration | 2,222 | 7 | 2,229 |  | 35,398 | 92 | 35,490 |  | 57,324 | 135 | 57,459 |
| (%) | 0.13 | 0.21 | 0.13 |  | 2.86 | 3.98 | 2.87 |  | 6.11 | 7.67 | 6.12 |
| Pit and fissure sealing | 627 | 0 | 627 |  | 4,163 | 10 | 4,173 |  | 3,543 | 4 | 3,547 |
| (%) | 0.04 | 0 | 0.04 |  | 0.34 | 0.43 | 0.34 |  | 0.38 | 0.23 | 0.38 |
| Caries, suspicious | 1,349 | 3 | 1,352 |  | 3,734 | 5 | 3,739 |  | 3,705 | 5 | 3,710 |
| (%) | 0.08 | 0.09 | 0.08 |  | 0.3 | 0.22 | 0.3 |  | 0.40 | 0.28 | 0.39 |
| Total | 1,744,096 | 3,270 | 1,747,366 |  | 1,236,064 | 2,313 | 1,238,377 |  | 937,851 | 1,760 | 939,611 |
| (%) | 100 | 100 | 100 |  | 100 | 100 | 100 |  | 100 | 100 | 100 |
| P | 0.000*** | | |  | 0.001** | | |  | 0.001** | | |

*P < 0.05, **P < 0.01, ***P < 0.001, CL/P: cleft lip with or without palate

**Supplementary Table 4. Comparison of tooth #53 between CL/P and non-CL/P children by age group**

| Age (months) | 18–29 | | |  | 42–53 | | |  | 54–65 | | |
| --- | --- | --- | --- | --- | --- | --- | --- | --- | --- | --- | --- |
| Tooth #53 | No CL/P | CL/P | Total |  | No CL/P | CL/P | Total |  | No CL/P | CL/P | Total |
| Eruption | 1,662,721 | 3,092 | 1,665,813 |  | 1,198,225 | 2,227 | 1,200,452 |  | 912,722 | 1,677 | 914,399 |
| (%) | 95.09 | 94.47 | 95.09 |  | 96.42 | 95.87 | 96.42 |  | 96.54 | 94.37 | 96.54 |
| Noneruption | 32,992 | 72 | 33,064 |  | 99 | 0 | 99 |  | 63 | 1 | 64 |
| (%) | 1.89 | 2.20 | 1.89 |  | 0.01 | 0.00 | 0.01 |  | 0.01 | 0.06 | 0.01 |
| Erupting | 33,371 | 64 | 33,435 |  | 255 | 0 | 255 |  | 183 | 1 | 184 |
| (%) | 1.91 | 1.96 | 1.91 |  | 0.02 | 0.00 | 0.02 |  | 0.02 | 0.06 | 0.02 |
| Demineralization | 7,094 | 13 | 7,107 |  | 5,257 | 5 | 5,262 |  | 2,417 | 6 | 2,423 |
| (%) | 0.41 | 0.40 | 0.41 |  | 0.42 | 0.22 | 0.42 |  | 0.26 | 0.34 | 0.26 |
| Caries | 11,253 | 30 | 11,283 |  | 30,651 | 73 | 30,724 |  | 20,359 | 64 | 20,423 |
| (%) | 0.64 | 0.92 | 0.64 |  | 2.47 | 3.14 | 2.47 |  | 2.15 | 3.60 | 2.16 |
| Restoration | 388 | 2 | 390 |  | 7,267 | 18 | 7,285 |  | 8,994 | 26 | 9,020 |
| (%) | 0.02 | 0.06 | 0.02 |  | 0.58 | 0.77 | 0.59 |  | 0.95 | 1.46 | 0.95 |
| Pit and fissure sealing | 108 | 0 | 108 |  | 14 | 0 | 14 |  | 20 | 0 | 20 |
| (%) | 0.01 | 0.00 | 0.01 |  | 0.00 | 0.00 | 0.00 |  | 0.00 | 0.00 | 0.00 |
| Caries, suspicious | 590 | 0 | 590 |  | 997 | 0 | 997 |  | 671 | 2 | 673 |
| (%) | 0.03 | 0.00 | 0.03 |  | 0.08 | 0.00 | 0.08 |  | 0.07 | 0.11 | 0.07 |
| Total | 1,748,517 | 3,273 | 1,751,790 |  | 1,242,765 | 2,323 | 1,245,088 |  | 945,429 | 1,777 | 947,206 |
| (%) | 100 | 100 | 100 |  | 100 | 100 | 100 |  | 100 | 100 | 100 |
| P | 0.240 | | |  | 0.151 | | |  | 0.000*** | | |

*P < 0.05, **P < 0.01, ***P < 0.001, CL/P: cleft lip with or without palate

**Supplementary Table 5. Comparison of tooth #52 between CL/P and non-CL/P children by age group**

| Age (months) | 18–29 | | |  | 42–53 | | |  | 54–65 | | |
| --- | --- | --- | --- | --- | --- | --- | --- | --- | --- | --- | --- |
| Tooth #52 | No CL/P | CL/P | Total |  | No CL/P | CL/P | Total |  | No CL/P | CL/P | Total |
| Eruption | 1,652,453 | 3,082 | 1,655,535 |  | 1,157,684 | 2,148 | 1,159,832 |  | 887,214 | 1,620 | 888,834 |
| (%) | 94.88 | 94.34 | 94.88 |  | 93.23 | 92.55 | 93.23 |  | 93.88 | 91.37 | 93.88 |
| Noneruption | 1,273 | 37 | 1,310 |  | 341 | 3 | 344 |  | 273 | 2 | 275 |
| (%) | 0.07 | 1.13 | 0.08 |  | 0.03 | 0.13 | 0.03 |  | 0.03 | 0.11 | 0.03 |
| Erupting | 1,273 | 6 | 1,279 |  | 239 | 0 | 239 |  | 172 | 0 | 172 |
| (%) | 0.07 | 0.18 | 0.07 |  | 0.02 | 0.00 | 0.02 |  | 0.02 | 0.00 | 0.02 |
| Demineralization | 21,630 | 45 | 21,675 |  | 6,341 | 9 | 6,350 |  | 2,972 | 4 | 2,976 |
| (%) | 1.24 | 1.38 | 1.24 |  | 0.51 | 0.39 | 0.51 |  | 0.31 | 0.23 | 0.31 |
| Caries | 61,275 | 91 | 61,366 |  | 62,278 | 131 | 62,409 |  | 42,100 | 107 | 42,207 |
| (%) | 3.52 | 2.79 | 3.52 |  | 5.02 | 5.64 | 5.02 |  | 4.45 | 6.03 | 4.46 |
| Restoration | 2,189 | 4 | 2,193 |  | 13,479 | 27 | 13,506 |  | 11,459 | 34 | 11,493 |
| (%) | 0.13 | 0.12 | 0.13 |  | 1.09 | 1.16 | 1.09 |  | 1.21 | 1.92 | 1.21 |
| Pit and fissure sealing | 47 | 0 | 47 |  | 19 | 0 | 19 |  | 20 | 0 | 20 |
| (%) | 0.00 | 0.00 | 0.00 |  | 0.00 | 0.00 | 0.00 |  | 0.00 | 0.00 | 0.00 |
| Caries, suspicious | 1,514 | 2 | 1,516 |  | 1,408 | 3 | 1,411 |  | 803 | 6 | 809 |
| (%) | 0.09 | 0.06 | 0.09 |  | 0.11 | 0.13 | 0.11 |  | 0.08 | 0.34 | 0.09 |
| Total | 1,741,654 | 3,267 | 1,744,921 |  | 1,241,789 | 2,321 | 1,244,110 |  | 945,013 | 1,773 | 946,786 |
| (%) | 100 | 100 | 100 |  | 100 | 100 | 100 |  | 100 | 100 | 100 |
| P | 0.000*** | | |  | 0.101 | | |  | 0.000*** | | |

*P < 0.05, **P < 0.01, ***P < 0.001, CL/P: cleft lip with or without palate

**Supplementary Table 6. Comparison of tooth #51 between CL/P and non-CL/P children by age group**

| Age (months) | 18–29 | | |  | 42–53 | | |  | 54–65 | | |
| --- | --- | --- | --- | --- | --- | --- | --- | --- | --- | --- | --- |
| Tooth #51 | No CL/P | CL/P | Total |  | No CL/P | CL/P | Total |  | No CL/P | CL/P | Total |
| Eruption | 1,608,961 | 3,031 | 1,611,992 |  | 1,058,984 | 1,978 | 1,060,962 |  | 818,682 | 1,518 | 820,200 |
| (%) | 92.73 | 93.15 | 92.73 |  | 85.61 | 85.37 | 85.61 |  | 86.88 | 85.57 | 86.87 |
| Noneruption | 368 | 3 | 371 |  | 538 | 2 | 540 |  | 619 | 2 | 621 |
| (%) | 0.02 | 0.09 | 0.02 |  | 0.04 | 0.09 | 0.04 |  | 0.07 | 0.11 | 0.07 |
| Erupting | 863 | 5 | 868 |  | 250 | 0 | 250 |  | 184 | 0 | 184 |
| (%) | 0.05 | 0.15 | 0.05 |  | 0.02 | 0.00 | 0.02 |  | 0.02 | 0.00 | 0.02 |
| Demineralization | 30,034 | 47 | 30,081 |  | 9,024 | 13 | 9,037 |  | 4,444 | 2 | 4,446 |
| (%) | 1.73 | 1.44 | 1.73 |  | 0.73 | 0.56 | 0.73 |  | 0.47 | 0.11 | 0.47 |
| Caries | 88,472 | 152 | 88,624 |  | 142,563 | 268 | 142,831 |  | 96,653 | 197 | 96,850 |
| (%) | 5.10 | 4.67 | 5.10 |  | 11.53 | 11.57 | 11.53 |  | 10.26 | 11.10 | 10.26 |
| Restoration | 3,168 | 10 | 3,178 |  | 22,551 | 47 | 22,598 |  | 19,982 | 49 | 20,031 |
| (%) | 0.18 | 0.31 | 0.18 |  | 1.82 | 2.03 | 1.82 |  | 2.12 | 2.76 | 2.12 |
| Pit and fissure sealing | 50 | 0 | 50 |  | 49 | 0 | 49 |  | 54 | 0 | 54 |
| (%) | 0.00 | 0.00 | 0.00 |  | 0.00 | 0.00 | 0.00 |  | 0.01 | 0.00 | 0.01 |
| Caries, suspicious | 3,114 | 6 | 3,120 |  | 2,994 | 9 | 3,003 |  | 1,732 | 6 | 1,738 |
| (%) | 0.18 | 0.18 | 0.18 |  | 0.24 | 0.39 | 0.24 |  | 0.18 | 0.34 | 0.18 |
| Total | 1,735,030 | 3,254 | 1,738,284 |  | 1,236,953 | 2,317 | 1,239,270 |  | 942,350 | 1,774 | 944,124 |
| (%) | 100 | 100 | 100 |  | 100 | 100 | 100 |  | 100 | 100 | 100 |
| P | 0.005** | | |  | 0.656 | | |  | 0.067 | | |

*P < 0.05, **P < 0.01, ***P < 0.001, CL/P: cleft lip with or without palate

**Supplementary Table 7. Comparison of tooth #61 between CL/P and non-CL/P children by age group**

| Age (months) | 18–29 | | |  | 42–53 | | |  | 54–65 | | |
| --- | --- | --- | --- | --- | --- | --- | --- | --- | --- | --- | --- |
| Tooth #61 | No CL/P | CL/P | Total |  | No CL/P | CL/P | Total |  | No CL/P | CL/P | Total |
| Eruption | 1,609,038 | 3,026 | 1,612,064 |  | 1,058,790 | 1,973 | 1,060,763 |  | 818,907 | 1510 | 820,417 |
| (%) | 92.74 | 92.94 | 92.74 |  | 85.60 | 85.37 | 85.60 |  | 86.90 | 84.97 | 86.89 |
| Noneruption | 361 | 5 | 366 |  | 534 | 2 | 536 |  | 600 | 1 | 601 |
| (%) | 0.02 | 0.15 | 0.02 |  | 0.04 | 0.09 | 0.04 |  | 0.06 | 0.06 | 0.06 |
| Erupting | 872 | 5 | 877 |  | 255 | 0 | 255 |  | 179 | 0 | 179 |
| (%) | 0.05 | 0.15 | 0.05 |  | 0.02 | 0.00 | 0.02 |  | 0.02 | 0.00 | 0.02 |
| Demineralization | 29,975 | 48 | 30,023 |  | 8,914 | 16 | 8,930 |  | 4,344 | 5 | 4,349 |
| (%) | 1.73 | 1.47 | 1.73 |  | 0.72 | 0.69 | 0.72 |  | 0.46 | 0.28 | 0.46 |
| Caries | 88,492 | 155 | 88,647 |  | 142,640 | 266 | 142,906 |  | 96,677 | 210 | 96,887 |
| (%) | 5.10 | 4.76 | 5.10 |  | 11.53 | 11.51 | 11.53 |  | 10.26 | 11.82 | 10.26 |
| Restoration | 3,125 | 10 | 3,135 |  | 22,704 | 46 | 22,750 |  | 19,881 | 45 | 19,926 |
| (%) | 0.18 | 0.31 | 0.18 |  | 1.84 | 1.99 | 1.84 |  | 2.11 | 2.53 | 2.11 |
| Pit and fissure sealing | 52 | 0 | 52 |  | 55 | 0 | 55 |  | 59 | 0 | 59 |
| (%) | 0.00 | 0.00 | 0.00 |  | 0.00 | 0.00 | 0.00 |  | 0.01 | 0.00 | 0.01 |
| Caries, suspicious | 3,167 | 7 | 3,174 |  | 3,023 | 8 | 3,031 |  | 1,734 | 6 | 1,740 |
| (%) | 0.18 | 0.21 | 0.18 |  | 0.24 | 0.35 | 0.24 |  | 0.18 | 0.34 | 0.18 |
| Total | 1,735,082 | 3,256 | 1,738,338 |  | 1,236,915 | 2,311 | 1,239,226 |  | 942,381 | 1777 | 944,158 |
| (%) | 100 | 100 | 100 |  | 100 | 100 | 100 |  | 100 | 100 | 100 |
| P | 0.000*** | | |  | 0.894 | | |  | 0.165 | | |

*P < 0.05, **P < 0.01, ***P < 0.001, CL/P cleft lip with or without palate

**Supplementary Table 8. Comparison of tooth #62 between CL/P and non-CL/P children by age group**

| Age (months) | 18–29 | | |  | 42–53 | | |  | 54–65 | | |
| --- | --- | --- | --- | --- | --- | --- | --- | --- | --- | --- | --- |
| Tooth #62 | No CL/P | CL/P | Total |  | No CL/P | CL/P | Total |  | No CL/P | CL/P | Total |
| Eruption | 1,651,362 | 3,068 | 1,654,430 |  | 1,155,709 | 2,121 | 1,157,830 |  | 885,646 | 1,615 | 887,261 |
| (%) | 94.83 | 93.85 | 94.83 |  | 93.08 | 91.54 | 93.08 |  | 93.73 | 90.99 | 93.72 |
| Noneruption | 1,219 | 54 | 1,273 |  | 357 | 5 | 362 |  | 256 | 0 | 256 |
| (%) | 0.07 | 1.65 | 0.07 |  | 0.03 | 0.22 | 0.03 |  | 0.03 | 0.00 | 0.03 |
| Erupting | 1,184 | 9 | 1,193 |  | 244 | 1 | 245 |  | 167 | 0 | 167 |
| (%) | 0.07 | 0.28 | 0.07 |  | 0.02 | 0.04 | 0.02 |  | 0.02 | 0.00 | 0.02 |
| Demineralization | 21,731 | 41 | 21,772 |  | 6,411 | 8 | 6,419 |  | 2,994 | 4 | 2,998 |
| (%) | 1.25 | 1.25 | 1.25 |  | 0.52 | 0.35 | 0.52 |  | 0.32 | 0.23 | 0.32 |
| Caries | 62,111 | 88 | 62,199 |  | 63,704 | 145 | 63,849 |  | 43,310 | 120 | 43,430 |
| (%) | 3.57 | 2.69 | 3.57 |  | 5.13 | 6.26 | 5.13 |  | 4.58 | 6.76 | 4.59 |
| Restoration | 2,182 | 6 | 2,188 |  | 13,713 | 34 | 13,747 |  | 11,682 | 34 | 11,716 |
| (%) | 0.13 | 0.18 | 0.13 |  | 1.10 | 1.47 | 1.11 |  | 1.24 | 1.92 | 1.24 |
| Pit and fissure sealing | 50 | 0 | 50 |  | 34 | 0 | 34 |  | 29 | 0 | 29 |
| (%) | 0.00 | 0.00 | 0.00 |  | 0.00 | 0.00 | 0.00 |  | 0.00 | 0.00 | 0.00 |
| Caries, suspicious | 1,551 | 3 | 1,554 |  | 1,427 | 3 | 1,430 |  | 856 | 2 | 858 |
| (%) | 0.09 | 0.09 | 0.09 |  | 0.11 | 0.13 | 0.11 |  | 0.09 | 0.11 | 0.09 |
| Total | 1,741,390 | 3,269 | 1,744,659 |  | 1,241,599 | 2,317 | 1,243,916 |  | 944,940 | 1,775 | 946,715 |
| (%) | 100 | 100 | 100 |  | 100 | 100 | 100 |  | 100 | 100 | 100 |
| P | 0.000*** | | |  | 0.000*** | | |  | 0.000*** | | |

*P < 0.05, **P < 0.01, ***P < 0.001, CL/P: cleft lip with or without palate

**Supplementary Table 9. Comparison of tooth #63 between CL/P and non-CL/P children by age group**

| Age (months) | 18–29 | | |  | 42–53 | | |  | 54–65 | | |
| --- | --- | --- | --- | --- | --- | --- | --- | --- | --- | --- | --- |
| Tooth #63 | No CL/P | CL/P | Total |  | No CL/P | CL/P | Total |  | No CL/P | CL/P | Total |
| Eruption | 1,662,809 | 3,098 | 1,665,907 |  | 1,198,352 | 2,227 | 1,200,579 |  | 913,129 | 1684 | 914,813 |
| (%) | 95.10 | 94.57 | 95.10 |  | 96.42 | 95.87 | 96.42 |  | 96.58 | 94.82 | 96.58 |
| Noneruption | 32,669 | 72 | 32,741 |  | 105 | 1 | 106 |  | 54 | 0 | 54 |
| (%) | 1.87 | 2.20 | 1.87 |  | 0.01 | 0.04 | 0.01 |  | 0.01 | 0.00 | 0.01 |
| Erupting | 33,267 | 69 | 33,336 |  | 247 | 0 | 247 |  | 180 | 2 | 182 |
| (%) | 1.90 | 2.11 | 1.90 |  | 0.02 | 0.00 | 0.02 |  | 0.02 | 0.11 | 0.02 |
| Demineralization | 7,186 | 15 | 7,201 |  | 5,242 | 5 | 5,247 |  | 2,398 | 5 | 2,403 |
| (%) | 0.41 | 0.46 | 0.41 |  | 0.42 | 0.22 | 0.42 |  | 0.25 | 0.28 | 0.25 |
| Caries | 11,522 | 19 | 11,541 |  | 30,604 | 72 | 30,676 |  | 20,066 | 55 | 20,121 |
| (%) | 0.66 | 0.58 | 0.66 |  | 2.46 | 3.10 | 2.46 |  | 2.12 | 3.10 | 2.12 |
| Restoration | 369 | 3 | 372 |  | 7,271 | 17 | 7,288 |  | 8,954 | 29 | 8,983 |
| (%) | 0.02 | 0.09 | 0.02 |  | 0.59 | 0.73 | 0.59 |  | 0.95 | 1.63 | 0.95 |
| Pit and fissure sealing | 108 | 0 | 108 |  | 25 | 0 | 25 |  | 24 | 0 | 24 |
| (%) | 0.01 | 0.00 | 0.01 |  | 0.00 | 0.00 | 0.00 |  | 0.00 | 0.00 | 0.00 |
| Caries, suspicious | 606 | 0 | 606 |  | 1,018 | 1 | 1,019 |  | 645 | 1 | 646 |
| (%) | 0.03 | 0.00 | 0.03 |  | 0.08 | 0.04 | 0.08 |  | 0.07 | 0.06 | 0.07 |
| Total | 1,748,536 | 3,276 | 1,751,812 |  | 1,242,864 | 2,323 | 1,245,187 |  | 945,450 | 1776 | 947,226 |
| (%) | 100 | 100 | 100 |  | 100 | 100 | 100 |  | 100 | 100 | 100 |
| P | 0.095 | | |  | 0.126 | | |  | 0.001** | | |

*P < 0.05, **P < 0.01, ***P < 0.001, CL/P: cleft lip with or without palate

**Supplementary Table 10. Comparison of tooth #64 between CL/P and non-CL/P children by age group**

| Age (months) | 18–29 | | |  | 42–53 | | |  | 54–65 | | |
| --- | --- | --- | --- | --- | --- | --- | --- | --- | --- | --- | --- |
| Tooth #64 | No CL/P | CL/P | Total |  | No CL/P | CL/P | Total |  | No CL/P | CL/P | Total |
| Eruption | 1,645,297 | 3,020 | 1,648,317 |  | 1,109,230 | 2,030 | 1,111,260 |  | 796,598 | 1451 | 798,049 |
| (%) | 94.34 | 92.47 | 94.34 |  | 89.76 | 87.65 | 89.76 |  | 84.98 | 82.40 | 84.98 |
| Noneruption | 16,056 | 43 | 16,099 |  | 98 | 1 | 99 |  | 114 | 0 | 114 |
| (%) | 0.92 | 1.32 | 0.92 |  | 0.01 | 0.04 | 0.01 |  | 0.01 | 0.00 | 0.01 |
| Erupting | 28,036 | 58 | 28,094 |  | 258 | 0 | 258 |  | 177 | 0 | 177 |
| (%) | 1.61 | 1.78 | 1.61 |  | 0.02 | 0.00 | 0.02 |  | 0.02 | 0.00 | 0.02 |
| Demineralization | 9,317 | 27 | 9,344 |  | 4,333 | 9 | 4,342 |  | 2,373 | 8 | 2,381 |
| (%) | 0.53 | 0.83 | 0.53 |  | 0.35 | 0.39 | 0.35 |  | 0.25 | 0.45 | 0.25 |
| Caries | 41,092 | 108 | 41,200 |  | 78,363 | 174 | 78,537 |  | 73,234 | 164 | 73,398 |
| (%) | 2.36 | 3.31 | 2.36 |  | 6.34 | 7.51 | 6.34 |  | 7.81 | 9.31 | 7.82 |
| Restoration | 2,172 | 8 | 2,180 |  | 35,390 | 85 | 35,475 |  | 57,577 | 127 | 57,704 |
| (%) | 0.12 | 0.24 | 0.12 |  | 2.86 | 3.67 | 2.87 |  | 6.14 | 7.21 | 6.14 |
| Pit and fissure sealing | 632 | 1 | 633 |  | 4,111 | 11 | 4,122 |  | 3,439 | 6 | 3,445 |
| (%) | 0.04 | 0.03 | 0.04 |  | 0.33 | 0.47 | 0.33 |  | 0.37 | 0.34 | 0.37 |
| Caries, suspicious | 1,384 | 1 | 1,385 |  | 3,983 | 6 | 3,989 |  | 3,865 | 5 | 3,870 |
| (%) | 0.08 | 0.03 | 0.08 |  | 0.32 | 0.26 | 0.32 |  | 0.41 | 0.28 | 0.41 |
| Total | 1,743,986 | 3,266 | 1,747,252 |  | 1,235,766 | 2,316 | 1,238,082 |  | 937,377 | 1761 | 939,138 |
| (%) | 100 | 100 | 100 |  | 100 | 100 | 100 |  | 100 | 100 | 100 |
| P | 0.000*** | | |  | 0.016* | | |  | 0.055 | | |

*P < 0.05, **P < 0.01, ***P < 0.001, CL/P: cleft lip with or without palate

**Supplementary Table 11. Comparison of tooth #65 between CL/P and non-CL/P children by age group**

| Age (months) | 18–29 | | |  | 42–53 | | |  | 54–65 | | |
| --- | --- | --- | --- | --- | --- | --- | --- | --- | --- | --- | --- |
| Tooth #65 | No CL/P | CL/P | Total |  | No CL/P | CL/P | Total |  | No CL/P | CL/P | Total |
| Eruption | 1,204,482 | 2,274 | 1,206,756 |  | 1,002,522 | 1,831 | 1,004,353 |  | 717,403 | 1292 | 718,695 |
| (%) | 68.29 | 68.91 | 68.29 |  | 81.67 | 80.20 | 81.67 |  | 76.96 | 73.83 | 76.96 |
| Noneruption | 518,947 | 937 | 519,884 |  | 1,140 | 2 | 1,142 |  | 471 | 0 | 471 |
| (%) | 29.42 | 28.39 | 29.42 |  | 0.09 | 0.09 | 0.09 |  | 0.05 | 0.00 | 0.05 |
| Erupting | 27,690 | 51 | 27,741 |  | 1,335 | 4 | 1,339 |  | 266 | 0 | 266 |
| (%) | 1.57 | 1.55 | 1.57 |  | 0.11 | 0.18 | 0.11 |  | 0.03 | 0.00 | 0.03 |
| Demineralization | 1,052 | 4 | 1,056 |  | 10,385 | 18 | 10,403 |  | 6,062 | 5 | 6,067 |
| (%) | 0.06 | 0.12 | 0.06 |  | 0.85 | 0.79 | 0.85 |  | 0.65 | 0.29 | 0.65 |
| Caries | 7,549 | 28 | 7,577 |  | 160,445 | 322 | 160,767 |  | 126,307 | 275 | 126,582 |
| (%) | 0.43 | 0.85 | 0.43 |  | 13.07 | 14.10 | 13.07 |  | 13.55 | 15.71 | 13.55 |
| Restoration | 2,133 | 4 | 2,137 |  | 41,738 | 92 | 41,830 |  | 72,833 | 166 | 72,999 |
| (%) | 0.12 | 0.12 | 0.12 |  | 3.40 | 4.03 | 3.40 |  | 7.81 | 9.49 | 7.82 |
| Pit and fissure sealing | 1,353 | 2 | 1,355 |  | 4,108 | 8 | 4,116 |  | 3,917 | 5 | 3,922 |
| (%) | 0.08 | 0.06 | 0.08 |  | 0.33 | 0.35 | 0.33 |  | 0.42 | 0.29 | 0.42 |
| Caries, suspicious | 546 | 0 | 546 |  | 5,812 | 6 | 5,818 |  | 4,892 | 7 | 4,899 |
| (%) | 0.03 | 0.00 | 0.03 |  | 0.47 | 0.26 | 0.47 |  | 0.52 | 0.40 | 0.52 |
| Total | 1,763,752 | 3,300 | 1,767,052 |  | 1,227,485 | 2,283 | 1,229,768 |  | 932,151 | 1750 | 933,901 |
| (%) | 100 | 100 | 100 |  | 100 | 100 | 100 |  | 100 | 100 | 100 |
| P | 0.011* | | |  | 0.307 | | |  | 0.004** | | |

*P < 0.05, **P < 0.01, ***P < 0.001, CL/P: cleft lip with or without palate

**Supplementary Table 12. Comparison of tooth #85 between CL/P and non-CL/P children by age group**

| Age (months) | 18–29 | | |  | 42–53 | | |  | 54–65 | | |
| --- | --- | --- | --- | --- | --- | --- | --- | --- | --- | --- | --- |
| Tooth #85 | No CL/P | CL/P | Total |  | No CL/P | CL/P | Total |  | No CL/P | CL/P | Total |
| Eruption | 1,216,760 | 2,302 | 1,219,062 |  | 926,487 | 1,703 | 928,190 |  | 677,267 | 1,233 | 678,500 |
| (%) | 69.10 | 69.76 | 69.10 |  | 75.70 | 74.82 | 75.69 |  | 72.71 | 70.62 | 72.71 |
| Noneruption | 475,104 | 892 | 475,996 |  | 741 | 3 | 744 |  | 320 | 0 | 320 |
| (%) | 26.98 | 27.03 | 26.98 |  | 0.06 | 0.13 | 0.06 |  | 0.03 | 0.00 | 0.03 |
| Erupting | 45,363 | 53 | 45,416 |  | 662 | 4 | 666 |  | 230 | 0 | 230 |
| (%) | 2.58 | 1.61 | 2.57 |  | 0.05 | 0.18 | 0.05 |  | 0.02 | 0.00 | 0.02 |
| Demineralization | 2,257 | 11 | 2,268 |  | 12,168 | 13 | 12,181 |  | 6,740 | 10 | 6,750 |
| (%) | 0.13 | 0.33 | 0.13 |  | 0.99 | 0.57 | 0.99 |  | 0.72 | 0.57 | 0.72 |
| Caries | 16,670 | 35 | 16,705 |  | 205,165 | 402 | 205,567 |  | 134,333 | 268 | 134,601 |
| (%) | 0.95 | 1.06 | 0.95 |  | 16.76 | 17.66 | 16.76 |  | 14.42 | 15.35 | 14.42 |
| Restoration | 2,230 | 4 | 2,234 |  | 67,499 | 130 | 67,629 |  | 103,521 | 218 | 103,739 |
| (%) | 0.13 | 0.12 | 0.13 |  | 5.51 | 5.71 | 5.52 |  | 11.11 | 12.49 | 11.12 |
| Pit and fissure sealing | 1,321 | 1 | 1,322 |  | 4,418 | 7 | 4,425 |  | 3,934 | 7 | 3,941 |
| (%) | 0.08 | 0.03 | 0.07 |  | 0.36 | 0.31 | 0.36 |  | 0.42 | 0.40 | 0.42 |
| Caries, suspicious | 1,102 | 2 | 1,104 |  | 6,825 | 14 | 6,839 |  | 5,061 | 10 | 5,071 |
| (%) | 0.06 | 0.06 | 0.06 |  | 0.56 | 0.62 | 0.56 |  | 0.54 | 0.57 | 0.54 |
| Total | 1,760,807 | 3,300 | 1,764,107 |  | 1,223,965 | 2,276 | 1,226,241 |  | 931,406 | 1,746 | 933,152 |
| (%) | 100 | 100 | 100 |  | 100 | 100 | 100 |  | 100 | 100 | 100 |
| P | 0.001** | | |  | 0.051 | | |  | 0.465 | | |

*P < 0.05, **P < 0.01, ***P < 0.001, CL/P: cleft lip with or without palate

**Supplementary Table 13. Comparison of tooth #84 between CL/P and non-CL/P children by age group**

| Age (months) | 18–29 | | |  | 42–53 | | |  | 54–65 | | |
| --- | --- | --- | --- | --- | --- | --- | --- | --- | --- | --- | --- |
| Tooth #84 | No CL/P | CL/P | Total |  | No CL/P | CL/P | Total |  | No CL/P | CL/P | Total |
| Eruption | 1,576,758 | 2,895 | 1,579,653 |  | 985,650 | 1,792 | 987,442 |  | 715,457 | 1,297 | 716,754 |
| (%) | 90.90 | 88.99 | 90.89 |  | 80.18 | 77.88 | 80.18 |  | 76.52 | 73.99 | 76.51 |
| Noneruption | 19,753 | 54 | 19,807 |  | 116 | 1 | 117 |  | 134 | 0 | 134 |
| (%) | 1.14 | 1.66 | 1.14 |  | 0.01 | 0.04 | 0.01 |  | 0.01 | 0.00 | 0.01 |
| Erupting | 21,933 | 46 | 21,979 |  | 265 | 0 | 265 |  | 181 | 1 | 182 |
| (%) | 1.26 | 1.41 | 1.26 |  | 0.02 | 0.00 | 0.02 |  | 0.02 | 0.06 | 0.02 |
| Demineralization | 14,477 | 34 | 14,511 |  | 6,831 | 9 | 6,840 |  | 3,655 | 4 | 3,659 |
| (%) | 0.83 | 1.05 | 0.83 |  | 0.56 | 0.39 | 0.56 |  | 0.39 | 0.23 | 0.39 |
| Caries | 93,079 | 204 | 93,283 |  | 156,912 | 323 | 157,235 |  | 108,749 | 224 | 108,973 |
| (%) | 5.37 | 6.27 | 5.37 |  | 12.77 | 14.04 | 12.77 |  | 11.63 | 12.78 | 11.63 |
| Restoration | 4,436 | 16 | 4,452 |  | 70,084 | 152 | 70,236 |  | 99,507 | 213 | 99,720 |
| (%) | 0.26 | 0.49 | 0.26 |  | 5.70 | 6.61 | 5.70 |  | 10.64 | 12.15 | 10.65 |
| Pit and fissure sealing | 595 | 0 | 595 |  | 3,642 | 12 | 3,654 |  | 3,006 | 6 | 3,012 |
| (%) | 0.03 | 0.00 | 0.03 |  | 0.30 | 0.52 | 0.30 |  | 0.32 | 0.34 | 0.32 |
| Caries, suspicious | 3,608 | 4 | 3,612 |  | 5,731 | 12 | 5,743 |  | 4,311 | 8 | 4,319 |
| (%) | 0.21 | 0.12 | 0.21 |  | 0.47 | 0.52 | 0.47 |  | 0.46 | 0.46 | 0.46 |
| Total | 1,734,639 | 3,253 | 1,737,892 |  | 1,229,231 | 2,301 | 1,231,532 |  | 935,000 | 1,753 | 936,753 |
| (%) | 100 | 100 | 100 |  | 100 | 100 | 100 |  | 100 | 100 | 100 |
| P | 0.001** | | |  | 0.023* | | |  | 0.193 | | |

*P < 0.05, **P < 0.01, ***P < 0.001, CL/P: cleft lip with or without palate

**Supplementary Table 14. Comparison of tooth #83 between CL/P and non-CL/P children by age group**

| Age (months) | 18–29 | | |  | 42–53 | | |  | 54–65 | | |
| --- | --- | --- | --- | --- | --- | --- | --- | --- | --- | --- | --- |
| Tooth #83 | No CL/P | CL/P | Total |  | No CL/P | CL/P | Total |  | No CL/P | CL/P | Total |
| Eruption | 1,668,941 | 3,085 | 1,672,026 |  | 1,216,693 | 2,270 | 1,218,963 |  | 922,347 | 1,713 | 924,060 |
| (%) | 95.35 | 94.08 | 95.35 |  | 97.79 | 97.51 | 97.79 |  | 97.49 | 96.29 | 97.49 |
| Noneruption | 42,711 | 102 | 42,813 |  | 361 | 1 | 362 |  | 190 | 0 | 190 |
| (%) | 2.44 | 3.11 | 2.44 |  | 0.03 | 0.04 | 0.03 |  | 0.02 | 0.00 | 0.02 |
| Erupting | 30,648 | 66 | 30,714 |  | 241 | 0 | 241 |  | 180 | 1 | 181 |
| (%) | 1.75 | 2.01 | 1.75 |  | 0.02 | 0.00 | 0.02 |  | 0.02 | 0.06 | 0.02 |
| Demineralization | 3,387 | 9 | 3,396 |  | 1,990 | 3 | 1,993 |  | 939 | 2 | 941 |
| (%) | 0.19 | 0.27 | 0.19 |  | 0.16 | 0.13 | 0.16 |  | 0.10 | 0.11 | 0.10 |
| Caries | 3,917 | 15 | 3,932 |  | 19,388 | 44 | 19,432 |  | 14,564 | 43 | 14,607 |
| (%) | 0.22 | 0.46 | 0.22 |  | 1.56 | 1.89 | 1.56 |  | 1.54 | 2.42 | 1.54 |
| Restoration | 260 | 2 | 262 |  | 4,633 | 8 | 4,641 |  | 7,334 | 16 | 7,350 |
| (%) | 0.01 | 0.06 | 0.01 |  | 0.37 | 0.34 | 0.37 |  | 0.78 | 0.90 | 0.78 |
| Pit and fissure sealing | 112 | 0 | 112 |  | 20 | 0 | 20 |  | 23 | 0 | 23 |
| (%) | 0.01 | 0.00 | 0.01 |  | 0.00 | 0.00 | 0.00 |  | 0.00 | 0.00 | 0.00 |
| Caries, suspicious | 329 | 0 | 329 |  | 831 | 2 | 833 |  | 512 | 4 | 516 |
| (%) | 0.02 | 0.00 | 0.02 |  | 0.07 | 0.09 | 0.07 |  | 0.05 | 0.22 | 0.05 |
| Total | 1,750,305 | 3,279 | 1,753,584 |  | 1,244,157 | 2,328 | 1,246,485 |  | 946,089 | 1,779 | 947,868 |
| (%) | 100 | 100 | 100 |  | 100 | 100 | 100 |  | 100 | 100 | 100 |
| P | 0.002** | | |  | 0.918 | | |  | 0.004** | | |

*P < 0.05, **P < 0.01, ***P < 0.001, CL/P: cleft lip with or without palate

**Supplementary Table 15. Comparison of tooth #82 between CL/P and non-CL/P children by age group**

| Age (months) | 18–29 | | |  | 42–53 | | |  | 54–65 | | |
| --- | --- | --- | --- | --- | --- | --- | --- | --- | --- | --- | --- |
| Tooth #82 | No CL/P | CL/P | Total |  | No CL/P | CL/P | Total |  | No CL/P | CL/P | Total |
| Eruption | 1,734,342 | 3,217 | 1,737,559 |  | 1,237,138 | 2,293 | 1,239,431 |  | 940,682 | 1,756 | 942,438 |
| (%) | 99.14 | 98.20 | 99.14 |  | 99.27 | 98.37 | 99.27 |  | 99.30 | 98.54 | 99.30 |
| Noneruption | 7,287 | 25 | 7,312 |  | 2,023 | 5 | 2,028 |  | 1,189 | 1 | 1,190 |
| (%) | 0.42 | 0.76 | 0.42 |  | 0.16 | 0.21 | 0.16 |  | 0.13 | 0.06 | 0.13 |
| Erupting | 1,708 | 7 | 1,715 |  | 248 | 0 | 248 |  | 189 | 0 | 189 |
| (%) | 0.10 | 0.21 | 0.10 |  | 0.02 | 0.00 | 0.02 |  | 0.02 | 0.00 | 0.02 |
| Demineralization | 2,619 | 8 | 2,627 |  | 748 | 2 | 750 |  | 382 | 0 | 382 |
| (%) | 0.15 | 0.24 | 0.15 |  | 0.06 | 0.09 | 0.06 |  | 0.04 | 0.00 | 0.04 |
| Caries | 3,071 | 18 | 3,089 |  | 5,471 | 29 | 5,500 |  | 4,365 | 24 | 4,389 |
| (%) | 0.18 | 0.55 | 0.18 |  | 0.44 | 1.24 | 0.44 |  | 0.46 | 1.35 | 0.46 |
| Restoration | 119 | 1 | 120 |  | 422 | 1 | 423 |  | 417 | 0 | 417 |
| (%) | 0.01 | 0.03 | 0.01 |  | 0.03 | 0.04 | 0.03 |  | 0.04 | 0.00 | 0.04 |
| Pit and fissure sealing | 62 | 0 | 62 |  | 12 | 0 | 12 |  | 18 | 0 | 18 |
| (%) | 0.00 | 0.00 | 0.00 |  | 0.00 | 0.00 | 0.00 |  | 0.00 | 0.00 | 0.00 |
| Caries, suspicious | 182 | 0 | 182 |  | 169 | 1 | 170 |  | 96 | 1 | 97 |
| (%) | 0.01 | 0.00 | 0.01 |  | 0.01 | 0.04 | 0.01 |  | 0.01 | 0.06 | 0.01 |
| Total | 1,749,390 | 3,276 | 1,752,666 |  | 1,246,231 | 2,331 | 1,248,562 |  | 947,338 | 1,782 | 949,120 |
| (%) | 100 | 100 | 100 |  | 100 | 100 | 100 |  | 100 | 100 | 100 |
| P | 0.000*** | | |  | 0.000*** | | |  | 0.000*** | | |

*P < 0.05, **P < 0.01, ***P < 0.001, CL/P: cleft lip with or without palate

**Supplementary Table 16. Comparison of tooth #81 between CL/P and non-CL/P children by age group**

| Age (months) | 18–29 | | |  | 42–53 | | |  | 54–65 | | |
| --- | --- | --- | --- | --- | --- | --- | --- | --- | --- | --- | --- |
| Tooth #81 | No CL/P | CL/P | Total |  | No CL/P | CL/P | Total |  | No CL/P | CL/P | Total |
| Eruption | 1,738,573 | 3,238 | 1,741,811 |  | 1,237,358 | 2,293 | 1,239,651 |  | 939,672 | 1,755 | 941,427 |
| (%) | 99.41 | 98.81 | 99.41 |  | 99.30 | 98.41 | 99.30 |  | 99.20 | 98.48 | 99.20 |
| Noneruption | 839 | 0 | 839 |  | 580 | 0 | 580 |  | 1,511 | 6 | 1,517 |
| (%) | 0.05 | 0.00 | 0.05 |  | 0.05 | 0.00 | 0.05 |  | 0.16 | 0.34 | 0.16 |
| Erupting | 796 | 5 | 801 |  | 245 | 0 | 245 |  | 234 | 0 | 234 |
| (%) | 0.05 | 0.15 | 0.05 |  | 0.02 | 0.00 | 0.02 |  | 0.02 | 0.00 | 0.02 |
| Demineralization | 3,394 | 9 | 3,403 |  | 832 | 2 | 834 |  | 461 | 1 | 462 |
| (%) | 0.19 | 0.27 | 0.19 |  | 0.07 | 0.09 | 0.07 |  | 0.05 | 0.06 | 0.05 |
| Caries | 4,816 | 24 | 4,840 |  | 6,555 | 33 | 6,588 |  | 4,976 | 20 | 4,996 |
| (%) | 0.28 | 0.73 | 0.28 |  | 0.53 | 1.42 | 0.53 |  | 0.53 | 1.12 | 0.53 |
| Restoration | 148 | 0 | 148 |  | 344 | 1 | 345 |  | 302 | 0 | 302 |
| (%) | 0.01 | 0.00 | 0.01 |  | 0.03 | 0.04 | 0.03 |  | 0.03 | 0.00 | 0.03 |
| Pit and fissure sealing | 42 | 0 | 42 |  | 12 | 0 | 12 |  | 11 | 0 | 11 |
| (%) | 0.00 | 0.00 | 0.00 |  | 0.00 | 0.00 | 0.00 |  | 0.00 | 0.00 | 0.00 |
| Caries, suspicious | 259 | 1 | 260 |  | 181 | 1 | 182 |  | 114 | 0 | 114 |
| (%) | 0.01 | 0.03 | 0.01 |  | 0.01 | 0.04 | 0.01 |  | 0.01 | 0.00 | 0.01 |
| Total | 1,748,867 | 3,277 | 1,752,144 |  | 1,246,107 | 2,330 | 1,248,437 |  | 947,281 | 1,782 | 949,063 |
| (%) | 100 | 100 | 100 |  | 100 | 100 | 100 |  | 100 | 100 | 100 |
| P | 0.000*** | | |  | 0.000*** | | |  | 0.018* | | |

*P < 0.05, **P < 0.01, ***P < 0.001, CL/P: cleft lip with or without palate

**Supplementary Table 17. Comparison of tooth #71 between CL/P and non-CL/P children by age group**

| Age (months) | 18–29 | | |  | 42–53 | | |  | 54–65 | | |
| --- | --- | --- | --- | --- | --- | --- | --- | --- | --- | --- | --- |
| Tooth #71 | No CL/P | CL/P | Total |  | No CL/P | CL/P | Total |  | No CL/P | CL/P | Total |
| Eruption | 1,738,625 | 3,236 | 1,741,861 |  | 1,237,479 | 2,296 | 1,239,775 |  | 939,801 | 1,754 | 941,555 |
| (%) | 99.41 | 98.78 | 99.41 |  | 99.31 | 98.50 | 99.31 |  | 99.21 | 98.43 | 99.21 |
| Noneruption | 720 | 1 | 721 |  | 527 | 0 | 527 |  | 1,418 | 5 | 1,423 |
| (%) | 0.04 | 0.03 | 0.04 |  | 0.04 | 0.00 | 0.04 |  | 0.15 | 0.28 | 0.15 |
| Erupting | 806 | 5 | 811 |  | 245 | 0 | 245 |  | 235 | 1 | 236 |
| (%) | 0.05 | 0.15 | 0.05 |  | 0.02 | 0.00 | 0.02 |  | 0.02 | 0.06 | 0.02 |
| Demineralization | 3,404 | 8 | 3,412 |  | 838 | 2 | 840 |  | 467 | 1 | 468 |
| (%) | 0.19 | 0.24 | 0.19 |  | 0.07 | 0.09 | 0.07 |  | 0.05 | 0.06 | 0.05 |
| Caries | 4,842 | 24 | 4,866 |  | 6,496 | 31 | 6,527 |  | 4,941 | 20 | 4,961 |
| (%) | 0.28 | 0.73 | 0.28 |  | 0.52 | 1.33 | 0.52 |  | 0.52 | 1.12 | 0.52 |
| Restoration | 157 | 1 | 158 |  | 345 | 1 | 346 |  | 289 | 1 | 290 |
| (%) | 0.01 | 0.03 | 0.01 |  | 0.03 | 0.04 | 0.03 |  | 0.03 | 0.06 | 0.03 |
| Pit and fissure sealing | 39 | 0 | 39 |  | 4 | 0 | 4 |  | 10 | 0 | 10 |
| (%) | 0.00 | 0.00 | 0.00 |  | 0.00 | 0.00 | 0.00 |  | 0.00 | 0.00 | 0.00 |
| Caries, suspicious | 265 | 1 | 266 |  | 183 | 1 | 184 |  | 115 | 0 | 115 |
| (%) | 0.02 | 0.03 | 0.02 |  | 0.01 | 0.04 | 0.01 |  | 0.01 | 0.00 | 0.01 |
| Total | 1,748,858 | 3,276 | 1,752,134 |  | 1,246,117 | 2,331 | 1,248,448 |  | 947,276 | 1,782 | 949,058 |
| (%) | 100 | 100 | 100 |  | 100 | 100 | 100 |  | 100 | 100 | 100 |
| P | 0.000*** | | |  | 0.000*** | | |  | 0.027* | | |

*P < 0.05, **P < 0.01, ***P < 0.001, CL/P: cleft lip with or without palate

**Supplementary Table 18. Comparison of tooth #72 between CL/P and non-CL/P children by age group**

| Age (months) | 18–29 | | |  | 42–53 | | |  | 54–65 | | |
| --- | --- | --- | --- | --- | --- | --- | --- | --- | --- | --- | --- |
| Tooth #72 | No CL/P | CL/P | Total |  | No CL/P | CL/P | Total |  | No CL/P | CL/P | Total |
| Eruption | 1,735,780 | 3,214 | 1,738,994 |  | 1,237,837 | 2,298 | 1,240,135 |  | 941,048 | 1,756 | 942,804 |
| (%) | 99.22 | 98.11 | 99.22 |  | 99.33 | 98.58 | 99.33 |  | 99.34 | 98.65 | 99.34 |
| Noneruption | 5,939 | 28 | 5,967 |  | 1,514 | 3 | 1,517 |  | 905 | 0 | 905 |
| (%) | 0.34 | 0.85 | 0.34 |  | 0.12 | 0.13 | 0.12 |  | 0.10 | 0.00 | 0.10 |
| Erupting | 1,635 | 6 | 1,641 |  | 247 | 0 | 247 |  | 187 | 0 | 187 |
| (%) | 0.09 | 0.18 | 0.09 |  | 0.02 | 0.00 | 0.02 |  | 0.02 | 0.00 | 0.02 |
| Demineralization | 2,652 | 10 | 2,662 |  | 726 | 1 | 727 |  | 382 | 0 | 382 |
| (%) | 0.15 | 0.31 | 0.15 |  | 0.06 | 0.04 | 0.06 |  | 0.04 | 0.00 | 0.04 |
| Caries | 2,979 | 17 | 2,996 |  | 5,346 | 27 | 5,373 |  | 4,282 | 23 | 4,305 |
| (%) | 0.17 | 0.52 | 0.17 |  | 0.43 | 1.16 | 0.43 |  | 0.45 | 1.29 | 0.45 |
| Restoration | 119 | 0 | 119 |  | 390 | 1 | 391 |  | 375 | 0 | 375 |
| (%) | 0.01 | 0.00 | 0.01 |  | 0.03 | 0.04 | 0.03 |  | 0.04 | 0.00 | 0.04 |
| Pit and fissure sealing | 56 | 0 | 56 |  | 13 | 0 | 13 |  | 21 | 0 | 21 |
| (%) | 0.00 | 0.00 | 0.00 |  | 0.00 | 0.00 | 0.00 |  | 0.00 | 0.00 | 0.00 |
| Caries, suspicious | 192 | 1 | 193 |  | 149 | 1 | 150 |  | 115 | 1 | 116 |
| (%) | 0.01 | 0.03 | 0.01 |  | 0.01 | 0.04 | 0.01 |  | 0.01 | 0.06 | 0.01 |
| Total | 1,749,352 | 3,276 | 1,752,628 |  | 1,246,222 | 2,331 | 1,248,553 |  | 947,315 | 1,780 | 949,095 |
| (%) | 100 | 100 | 100 |  | 100 | 100 | 100 |  | 100 | 100 | 100 |
| P | 0.000*** | | |  | 0.000*** | | |  | 0.000*** | | |

*P < 0.05, **P < 0.01, ***P < 0.001, CL/P: cleft lip with or without palate

**Supplementary Table 19. Comparison of tooth #73 between CL/P and non-CL/P children by age group**

| Age (months) | 18–29 | | |  | 42–53 | | |  | 54–65 | | |
| --- | --- | --- | --- | --- | --- | --- | --- | --- | --- | --- | --- |
| Tooth #73 | No CL/P | CL/P | Total |  | No CL/P | CL/P | Total |  | No CL/P | CL/P | Total |
| Eruption | 1,669,539 | 3,082 | 1,672,621 |  | 1,215,994 | 2,271 | 1,218,265 |  | 922,044 | 1,715 | 923,759 |
| (%) | 95.38 | 93.99 | 95.38 |  | 97.74 | 97.59 | 97.74 |  | 97.47 | 96.29 | 97.46 |
| Noneruption | 42,655 | 103 | 42,758 |  | 305 | 1 | 306 |  | 148 | 0 | 148 |
| (%) | 2.44 | 3.14 | 2.44 |  | 0.02 | 0.04 | 0.02 |  | 0.02 | 0.00 | 0.02 |
| Erupting | 30,385 | 69 | 30,454 |  | 237 | 0 | 237 |  | 186 | 1 | 187 |
| (%) | 1.74 | 2.10 | 1.74 |  | 0.02 | 0.00 | 0.02 |  | 0.02 | 0.06 | 0.02 |
| Demineralization | 3,376 | 8 | 3,384 |  | 2,117 | 1 | 2,118 |  | 951 | 0 | 951 |
| (%) | 0.19 | 0.24 | 0.19 |  | 0.17 | 0.04 | 0.17 |  | 0.10 | 0.00 | 0.10 |
| Caries | 3,719 | 14 | 3,733 |  | 19,970 | 41 | 20,011 |  | 14,578 | 37 | 14,615 |
| (%) | 0.21 | 0.43 | 0.21 |  | 1.61 | 1.76 | 1.61 |  | 1.54 | 2.08 | 1.54 |
| Restoration | 230 | 3 | 233 |  | 4,714 | 11 | 4,725 |  | 7,552 | 23 | 7,575 |
| (%) | 0.01 | 0.09 | 0.01 |  | 0.38 | 0.47 | 0.38 |  | 0.80 | 1.29 | 0.80 |
| Pit and fissure sealing | 117 | 0 | 117 |  | 19 | 0 | 19 |  | 21 | 0 | 21 |
| (%) | 0.01 | 0.00 | 0.01 |  | 0.00 | 0.00 | 0.00 |  | 0.00 | 0.00 | 0.00 |
| Caries, suspicious | 311 | 0 | 311 |  | 810 | 2 | 812 |  | 541 | 5 | 546 |
| (%) | 0.02 | 0.00 | 0.02 |  | 0.07 | 0.09 | 0.07 |  | 0.06 | 0.28 | 0.06 |
| Total | 1,750,332 | 3,279 | 1,753,611 |  | 1,244,166 | 2,327 | 1,246,493 |  | 946,021 | 1,781 | 947,802 |
| (%) | 100 | 100 | 100 |  | 100 | 100 | 100 |  | 100 | 100 | 100 |
| P | 0.000*** | | |  | 0.772 | | |  | 0.000*** | | |

*P < 0.05, **P < 0.01, ***P < 0.001, CL/P: cleft lip with or without palate

**Supplementary Table 20. Comparison of tooth #74 between CL/P and non-CL/P children by age group**

| Age (months) | 18–29 | | |  | 42–53 | | |  | 54–65 | | |
| --- | --- | --- | --- | --- | --- | --- | --- | --- | --- | --- | --- |
| Tooth #74 | No CL/P | CL/P | Total |  | No CL/P | CL/P | Total |  | No CL/P | CL/P | Total |
| Eruption | 1,587,428 | 2,927 | 1,590,355 |  | 996,547 | 1,843 | 998,390 |  | 722,952 | 1,307 | 724,259 |
| (%) | 91.47 | 89.98 | 91.47 |  | 81.01 | 79.92 | 81.01 |  | 77.28 | 74.30 | 77.28 |
| Noneruption | 19,740 | 44 | 19,784 |  | 114 | 1 | 115 |  | 138 | 0 | 138 |
| (%) | 1.14 | 1.35 | 1.14 |  | 0.01 | 0.04 | 0.01 |  | 0.01 | 0.00 | 0.01 |
| Erupting | 22,090 | 47 | 22,137 |  | 270 | 0 | 270 |  | 189 | 1 | 190 |
| (%) | 1.27 | 1.44 | 1.27 |  | 0.02 | 0.00 | 0.02 |  | 0.02 | 0.06 | 0.02 |
| Demineralization | 14,057 | 36 | 14,093 |  | 6,846 | 11 | 6,857 |  | 3,476 | 4 | 3,480 |
| (%) | 0.81 | 1.11 | 0.81 |  | 0.56 | 0.48 | 0.56 |  | 0.37 | 0.23 | 0.37 |
| Caries | 84,168 | 184 | 84,352 |  | 149,663 | 288 | 149,951 |  | 103,985 | 227 | 104,212 |
| (%) | 4.85 | 5.66 | 4.85 |  | 12.17 | 12.49 | 12.17 |  | 11.12 | 12.91 | 11.12 |
| Restoration | 4,037 | 13 | 4,050 |  | 67,438 | 145 | 67,583 |  | 97,496 | 201 | 97,697 |
| (%) | 0.23 | 0.40 | 0.23 |  | 5.48 | 6.29 | 5.48 |  | 10.42 | 11.43 | 10.42 |
| Pit and fissure sealing | 612 | 0 | 612 |  | 3,659 | 11 | 3,670 |  | 3,054 | 6 | 3,060 |
| (%) | 0.04 | 0.00 | 0.04 |  | 0.30 | 0.48 | 0.30 |  | 0.33 | 0.34 | 0.33 |
| Caries, suspicious | 3,319 | 2 | 3,321 |  | 5,596 | 7 | 5,603 |  | 4,174 | 13 | 4,187 |
| (%) | 0.19 | 0.06 | 0.19 |  | 0.45 | 0.30 | 0.45 |  | 0.45 | 0.74 | 0.45 |
| Total | 1,735,451 | 3,253 | 1,738,704 |  | 1,230,133 | 2,306 | 1,232,439 |  | 935,464 | 1,759 | 937,223 |
| (%) | 100 | 100 | 100 |  | 100 | 100 | 100 |  | 100 | 100 | 100 |
| P | 0.009** | | |  | 0.160 | | |  | 0.042* | | |

*P < 0.05, **P < 0.01, ***P < 0.001, CL/P: cleft lip with or without palate

**Supplementary Table 21. Comparison of tooth #75 between CL/P and non-CL/P children by age group**

| Age (months) | 18–29 | | |  | 42–53 | | |  | 54–65 | | |
| --- | --- | --- | --- | --- | --- | --- | --- | --- | --- | --- | --- |
| Tooth #75 | No CL/P | CL/P | Total |  | No CL/P | CL/P | Total |  | No CL/P | CL/P | Total |
| Eruption | 1,217,781 | 2,315 | 1,220,096 |  | 918,326 | 1,718 | 920,044 |  | 672,900 | 1,216 | 674,116 |
| (%) | 69.18 | 70.19 | 69.18 |  | 75.08 | 75.22 | 75.08 |  | 72.28 | 69.76 | 72.27 |
| Noneruption | 468,643 | 878 | 469,521 |  | 757 | 2 | 759 |  | 382 | 0 | 382 |
| (%) | 26.62 | 26.62 | 26.62 |  | 0.06 | 0.09 | 0.06 |  | 0.04 | 0.00 | 0.04 |
| Erupting | 48,342 | 53 | 48,395 |  | 650 | 2 | 652 |  | 235 | 0 | 235 |
| (%) | 2.75 | 1.61 | 2.74 |  | 0.05 | 0.09 | 0.05 |  | 0.03 | 0.00 | 0.03 |
| Demineralization | 2,626 | 8 | 2,634 |  | 12,988 | 18 | 13,006 |  | 7,084 | 9 | 7,093 |
| (%) | 0.15 | 0.24 | 0.15 |  | 1.06 | 0.79 | 1.06 |  | 0.76 | 0.52 | 0.76 |
| Caries | 18,099 | 39 | 18,138 |  | 209,958 | 383 | 210,341 |  | 136,320 | 288 | 136,608 |
| (%) | 1.03 | 1.18 | 1.03 |  | 17.17 | 16.77 | 17.16 |  | 14.64 | 16.52 | 14.65 |
| Restoration | 2,271 | 3 | 2,274 |  | 69,116 | 139 | 69,255 |  | 105,028 | 214 | 105,242 |
| (%) | 0.13 | 0.09 | 0.13 |  | 5.65 | 6.09 | 5.65 |  | 11.28 | 12.28 | 11.28 |
| Pit and fissure sealing | 1,355 | 1 | 1,356 |  | 4,342 | 8 | 4,350 |  | 3,868 | 5 | 3,873 |
| (%) | 0.08 | 0.03 | 0.08 |  | 0.35 | 0.35 | 0.35 |  | 0.42 | 0.29 | 0.42 |
| Caries, suspicious | 1,237 | 1 | 1,238 |  | 6,994 | 14 | 7,008 |  | 5,210 | 11 | 5,221 |
| (%) | 0.07 | 0.03 | 0.07 |  | 0.57 | 0.61 | 0.57 |  | 0.56 | 0.63 | 0.56 |
| Total | 1,760,354 | 3,298 | 1,763,652 |  | 1,223,131 | 2,284 | 1,225,415 |  | 931,027 | 1,743 | 932,770 |
| (%) | 100 | 100 | 100 |  | 100 | 100 | 100 |  | 100 | 100 | 100 |
| P | 0.004** | | |  | 0.845 | | |  | 0.156 | | |

*P < 0.05, **P < 0.01, ***P < 0.001, CL/P: cleft lip with or without palate

**Supplementary Table 22. Comparison of permanent teeth at 54–65 months (Right upper)**

| Permanent teeth | #16 | | |  | #12 | | |  | #11 | | |
| --- | --- | --- | --- | --- | --- | --- | --- | --- | --- | --- | --- |
| 54–65 months | No CL/P | CL/P | Total |  | No CL/P | CL/P | Total |  | No CL/P | CL/P | Total |
| Eruption | 728,134 | 1,420 | 729,554 |  | 762,971 | 1,482 | 764,453 |  | 759,984 | 1,476 | 761,460 |
| (%) | 76.18 | 79.33 | 76.19 |  | 79.93 | 82.84 | 79.94 |  | 79.65 | 82.60 | 79.65 |
| Noneruption | 223,560 | 365 | 223,925 |  | 188,237 | 299 | 188,536 |  | 187,679 | 299 | 187,978 |
| (%) | 23.39 | 20.39 | 23.38 |  | 19.72 | 16.71 | 19.72 |  | 19.67 | 16.73 | 19.66 |
| Erupting | 1,129 | 0 | 1,129 |  | 163 | 1 | 164 |  | 185 | 1 | 186 |
| (%) | 0.12 | 0.00 | 0.12 |  | 0.02 | 0.06 | 0.02 |  | 0.02 | 0.06 | 0.02 |
| Demineralization | 47 | 1 | 48 |  | 167 | 0 | 167 |  | 173 | 0 | 173 |
| (%) | 0.00 | 0.06 | 0.01 |  | 0.02 | 0.00 | 0.02 |  | 0.02 | 0.00 | 0.02 |
| Caries | 955 | 2 | 957 |  | 1,550 | 4 | 1,554 |  | 4,581 | 9 | 4,590 |
| (%) | 0.10 | 0.11 | 0.10 |  | 0.16 | 0.22 | 0.16 |  | 0.48 | 0.50 | 0.48 |
| Restoration | 1,399 | 1 | 1,400 |  | 1,075 | 1 | 1,076 |  | 1,285 | 1 | 1,286 |
| (%) | 0.15 | 0.06 | 0.15 |  | 0.11 | 0.06 | 0.11 |  | 0.13 | 0.06 | 0.13 |
| Pit and fissure sealing | 493 | 0 | 493 |  | 256 | 1 | 257 |  | 82 | 0 | 82 |
| (%) | 0.05 | 0.00 | 0.05 |  | 0.03 | 0.06 | 0.03 |  | 0.01 | 0.00 | 0.01 |
| Caries, suspicious | 69 | 1 | 70 |  | 86 | 1 | 87 |  | 192 | 1 | 193 |
| (%) | 0.01 | 0.06 | 0.01 |  | 0.01 | 0.06 | 0.01 |  | 0.02 | 0.06 | 0.02 |
| Total | 955,786 | 1,790 | 957,576 |  | 954,505 | 1,789 | 956,294 |  | 954,161 | 1,787 | 955,948 |
| (%) | 100 | 100 | 100 |  | 100 | 100 | 100 |  | 100 | 100 | 100 |
| P | 0.000*** | | |  | 0.013* | | |  | 0.062 | | |

*P < 0.05, **P < 0.01, ***P < 0.001, CL/P: cleft lip with or without palate

**Supplementary Table 23. Comparison of permanent teeth at 54–65 months (Left upper)**

| Permanent teeth | #21 | | |  | #22 | | |  | #26 | | |
| --- | --- | --- | --- | --- | --- | --- | --- | --- | --- | --- | --- |
| 54–65 months | No CL/P | CL/P | Total |  | No CL/P | CL/P | Total |  | No CL/P | CL/P | Total |
| Eruption | 760,218 | 1,478 | 761,696 |  | 763,545 | 1,480 | 765,025 |  | 728,908 | 1,422 | 730,330 |
| (%) | 79.67 | 82.71 | 79.68 |  | 79.99 | 82.77 | 80.00 |  | 76.27 | 79.44 | 76.28 |
| Noneruption | 187,548 | 298 | 187,846 |  | 188,053 | 300 | 188,353 |  | 221,775 | 363 | 222,138 |
| (%) | 19.66 | 16.68 | 19.65 |  | 19.70 | 16.78 | 19.70 |  | 23.21 | 20.28 | 23.20 |
| Erupting | 175 | 1 | 176 |  | 334 | 1 | 335 |  | 1,252 | 0 | 1,252 |
| (%) | 0.02 | 0.06 | 0.02 |  | 0.03 | 0.06 | 0.04 |  | 0.13 | 0.00 | 0.13 |
| Demineralization | 166 | 0 | 166 |  | 106 | 0 | 106 |  | 71 | 0 | 71 |
| (%) | 0.02 | 0.00 | 0.02 |  | 0.01 | 0.00 | 0.01 |  | 0.01 | 0.00 | 0.01 |
| Caries | 4,501 | 8 | 4,509 |  | 1,385 | 5 | 1,390 |  | 1,437 | 2 | 1,439 |
| (%) | 0.47 | 0.45 | 0.47 |  | 0.15 | 0.28 | 0.15 |  | 0.15 | 0.11 | 0.15 |
| Restoration | 1,267 | 1 | 1,268 |  | 978 | 1 | 979 |  | 1,620 | 2 | 1,622 |
| (%) | 0.13 | 0.06 | 0.13 |  | 0.10 | 0.06 | 0.10 |  | 0.17 | 0.11 | 0.17 |
| Pit and fissure sealing | 81 | 0 | 81 |  | 78 | 0 | 78 |  | 535 | 0 | 535 |
| (%) | 0.01 | 0.00 | 0.01 |  | 0.01 | 0.00 | 0.01 |  | 0.06 | 0.00 | 0.06 |
| Caries, suspicious | 197 | 1 | 198 |  | 70 | 1 | 71 |  | 87 | 1 | 88 |
| (%) | 0.02 | 0.06 | 0.02 |  | 0.01 | 0.06 | 0.01 |  | 0.01 | 0.06 | 0.01 |
| Total | 954,153 | 1,787 | 955,940 |  | 954,549 | 1,788 | 956,337 |  | 955,685 | 1,790 | 957,475 |
| (%) | 100 | 100 | 100 |  | 100 | 100 | 100 |  | 100 | 100 | 100 |
| P | 0.054 | | |  | 0.011* | | |  | 0.016* | | |

*P < 0.05, **P < 0.01, ***P < 0.001, CL/P: cleft lip with or without palate

**Supplementary Table 24. Comparison of permanent teeth at 54–65 months (Right lower)**

| Permanent teeth | #46 | | |  | #42 | | |  | #41 | | |
| --- | --- | --- | --- | --- | --- | --- | --- | --- | --- | --- | --- |
| 54–65 months | No CL/P | CL/P | Total |  | No CL/P | CL/P | Total |  | No CL/P | CL/P | Total |
| Eruption | 728,038 | 1,418 | 729,456 |  | 768,326 | 1,489 | 769,815 |  | 768,865 | 1,490 | 770,355 |
| (%) | 76.19 | 79.17 | 76.19 |  | 80.49 | 83.23 | 80.50 |  | 80.55 | 83.29 | 80.56 |
| Noneruption | 220,396 | 361 | 220,757 |  | 184,452 | 293 | 184,745 |  | 182,926 | 292 | 183,218 |
| (%) | 23.06 | 20.16 | 23.06 |  | 19.32 | 16.38 | 19.32 |  | 19.17 | 16.32 | 19.16 |
| Erupting | 2,993 | 4 | 2,997 |  | 520 | 2 | 522 |  | 1,382 | 3 | 1,385 |
| (%) | 0.31 | 0.22 | 0.31 |  | 0.05 | 0.11 | 0.05 |  | 0.14 | 0.17 | 0.14 |
| Demineralization | 82 | 0 | 82 |  | 24 | 1 | 25 |  | 37 | 1 | 38 |
| (%) | 0.01 | 0.00 | 0.01 |  | 0.00 | 0.06 | 0.00 |  | 0.00 | 0.06 | 0.00 |
| Caries | 1,695 | 3 | 1,698 |  | 354 | 2 | 356 |  | 419 | 1 | 420 |
| (%) | 0.18 | 0.17 | 0.18 |  | 0.04 | 0.11 | 0.04 |  | 0.04 | 0.06 | 0.04 |
| Restoration | 1,580 | 3 | 1,583 |  | 747 | 1 | 748 |  | 738 | 1 | 739 |
| (%) | 0.17 | 0.17 | 0.17 |  | 0.08 | 0.06 | 0.08 |  | 0.08 | 0.06 | 0.08 |
| Pit and fissure sealing | 673 | 1 | 674 |  | 74 | 0 | 74 |  | 79 | 0 | 79 |
| (%) | 0.07 | 0.06 | 0.07 |  | 0.01 | 0.00 | 0.01 |  | 0.01 | 0.00 | 0.01 |
| Caries, suspicious | 116 | 1 | 117 |  | 28 | 1 | 29 |  | 30 | 1 | 31 |
| (%) | 0.01 | 0.06 | 0.01 |  | 0.00 | 0.06 | 0.00 |  | 0.00 | 0.06 | 0.00 |
| Total | 955,573 | 1,791 | 957,364 |  | 954,525 | 1,789 | 956,314 |  | 954,476 | 1,789 | 956,265 |
| (%) | 100 | 100 | 100 |  | 100 | 100 | 100 |  | 100 | 100 | 100 |
| P | 0.097 | | |  | 0.000*** | | |  | 0.000*** | | |

*P < 0.05, **P < 0.01, ***P < 0.001, CL/P: cleft lip with or without palate

**Supplementary Table 25. Comparison of permanent teeth at 54–65 months (Left lower)**

| Permanent teeth | #31 | | |  | #32 | | |  | #36 | | |
| --- | --- | --- | --- | --- | --- | --- | --- | --- | --- | --- | --- |
| 54–65 months | No CL/P | CL/P | Total |  | No CL/P | CL/P | Total |  | No CL/P | CL/P | Total |
| Eruption | 769,247 | 1,489 | 770,736 |  | 768,319 | 1,488 | 769,807 |  | 728,584 | 1,419 | 730,003 |
| (%) | 80.60 | 83.23 | 80.60 |  | 80.49 | 83.17 | 80.50 |  | 76.25 | 79.27 | 76.25 |
| Noneruption | 182,306 | 291 | 182,597 |  | 184,644 | 296 | 184,940 |  | 218,623 | 358 | 218,981 |
| (%) | 19.10 | 16.27 | 19.10 |  | 19.34 | 16.55 | 19.34 |  | 22.88 | 20.00 | 22.87 |
| Erupting | 1,606 | 5 | 1,611 |  | 326 | 1 | 327 |  | 3,307 | 3 | 3,310 |
| (%) | 0.17 | 0.28 | 0.17 |  | 0.03 | 0.06 | 0.03 |  | 0.35 | 0.17 | 0.35 |
| Demineralization | 33 | 1 | 34 |  | 33 | 1 | 34 |  | 127 | 0 | 127 |
| (%) | 0.00 | 0.06 | 0.00 |  | 0.00 | 0.06 | 0.00 |  | 0.01 | 0.00 | 0.01 |
| Caries | 416 | 2 | 418 |  | 353 | 1 | 354 |  | 2,188 | 5 | 2,193 |
| (%) | 0.04 | 0.11 | 0.04 |  | 0.04 | 0.06 | 0.04 |  | 0.23 | 0.28 | 0.23 |
| Restoration | 741 | 1 | 742 |  | 749 | 1 | 750 |  | 1,813 | 2 | 1,815 |
| (%) | 0.08 | 0.06 | 0.08 |  | 0.08 | 0.06 | 0.08 |  | 0.19 | 0.11 | 0.19 |
| Pit and fissure sealing | 76 | 0 | 76 |  | 80 | 0 | 80 |  | 752 | 2 | 754 |
| (%) | 0.01 | 0.00 | 0.01 |  | 0.01 | 0.00 | 0.01 |  | 0.08 | 0.11 | 0.08 |
| Caries, suspicious | 30 | 0 | 30 |  | 26 | 1 | 27 |  | 139 | 1 | 140 |
| (%) | 0.00 | 0.00 | 0.00 |  | 0.00 | 0.06 | 0.00 |  | 0.01 | 0.06 | 0.01 |
| Total | 954,455 | 1,789 | 956,244 |  | 954,530 | 1,789 | 956,319 |  | 955,533 | 1,790 | 957,323 |
| (%) | 100 | 100 | 100 |  | 100 | 100 | 100 |  | 100 | 100 | 100 |
| P | 0.000*** | | |  | 0.000*** | | |  | 0.059 | | |

*P < 0.05, **P < 0.01, ***P < 0.001, CL/P: cleft lip with or without palate

**Supplementary Table 26. Statistical differences between CL/P and non-CL/P children by age group and each tooth.**

| Age (months) | Number of teeth | | | | | | | | | | | |
| --- | --- | --- | --- | --- | --- | --- | --- | --- | --- | --- | --- | --- |
| 18–29 |  | 55 | 54 | 53 | 52 | 51 | 61 | 62 | 63 | 64 | 65 |  |
|  |  | * | *** |  | *** | ** | *** | *** |  | *** | * |  |
|  |  | 85 | 84 | 83 | 82 | 81 | 71 | 72 | 73 | 74 | 75 |  |
|  |  | ** | ** | ** | *** | *** | *** | *** | *** | ** | ** |  |
| 42–53 |  | 55 | 54 | 53 | 52 | 51 | 61 | 62 | 63 | 64 | 65 |  |
|  |  |  | ** |  |  |  |  | *** |  | * |  |  |
|  |  | 85 | 84 | 83 | 82 | 81 | 71 | 72 | 73 | 74 | 75 |  |
|  |  |  | * |  | *** | *** | *** | *** |  |  |  |  |
| 54–65 | 16 |  |  |  | 12 | 11 | 21 | 22 |  |  |  | 26 |
|  | *** |  |  |  | * |  |  | * |  |  |  | * |
|  |  | 55 | 54 | 53 | 52 | 51 | 61 | 62 | 63 | 64 | 65 |  |
|  |  | ** | ** | *** | *** |  |  | *** | ** |  | ** |  |
|  |  | 85 | 84 | 83 | 82 | 81 | 71 | 72 | 73 | 74 | 75 |  |
|  |  |  |  | ** | *** | * | * | *** | *** | * |  |  |
|  | 46 |  |  |  | 42 | 41 | 31 | 32 |  |  |  | 36 |
|  |  |  |  |  | *** | *** | *** | *** |  |  |  |  |

*P < 0.05, **P < 0.01, ***P < 0.001
